# Comprehensive Assessment and Potential Ecological Risk of Trace Element Pollution (As, Ni, Co and Cr) in Aquatic Environmental Samples from an Industrialized Area

**DOI:** 10.3390/ijerph18147348

**Published:** 2021-07-09

**Authors:** M. Díaz-de-Alba, M. D. Granado-Castro, M. D. Galindo-Riaño, M. J. Casanueva-Marenco

**Affiliations:** Department of Analytical Chemistry, Institute of Biomolecules (INBIO), Faculty of Sciences, CEI-MAR, Campus Río San Pedro, University of Cádiz, ES-11510 Puerto Real, Spain; margarita.diaz@uca.es (M.D.-d.-A.); dolores.galindo@uca.es (M.D.G.-R.); mariajose.casanueva@uca.es (M.J.C.-M.)

**Keywords:** metal pollution, sediment, water, fish, toxicity, bioavailability, speciation

## Abstract

A global assessment of arsenic (As), nickel (Ni), cobalt (Co) and chromium (Cr) was performed in environmental samples from an important industrial bay. Different fractions of water, sediments and tissues from four species of fish were analysed. Samples were collected from selected sampling sites during four consecutive samplings in spring and autumn seasons, in order to evaluate concentrations and their possible correlations among the aquatic compartments. While a higher availability of Cr and Ni was found in water, Co and As were the most available elements in sediments. In fish, the liver was the tissue with the highest proportion of As and Co, and gills showed the highest concentrations of Ni and Cr. Significance differences were observed among sites showing the pollution sources. In sediments, high correlations were found between total Co content and the most available fractions. Total Ni content highly correlated with the oxidisable fraction, while Cr total content tightly correlated with the least available fractions. Quality guideline values for sediments were frequently exceeded. In sediments and biota, concentrations were slightly higher than in other ecosystems, indicating that maritime, industrial and urban activities are affecting this type of ecosystem with great anthropogenic influence.

## 1. Introduction

Ongoing meteorological processes and biological activities, such as rainfall or soil erosion, as well as human actions on the environment, such as drainage and sewage disposal, play an important role in the distribution of different semimetals and metals in aquatic ecosystems [1,2]. Metal and semimetal contamination of the aquatic environment may lead to unsuitable water for ecosystem biodiversity or supporting aquaculture, and may increase frequency of diseases [3,4]. Furthermore, these elements tend to accumulate in living organisms, such as fish and invertebrates, posing a risk to predators and humans through the biomagnification process [5,6,7].

Coastal areas adjacent to extensive industrial and urban territories have higher metal and semimetal levels and, therefore, deserve much attention because of their potential ecological effects and public concern for seafood safety [8,9,10]. Thus, it is necessary to monitor the current pollution status of these elements in coastal ecosystems in order to ensure public health and the sustainable development of marine ecosystems and aiding coastal management decisions [11,12]. How metals and semimetals behave in the aquatic environment will depend on their chemical composition, as this will affect how they are distributed and mobilized in the environment, their level of toxicity and their bioavailability. Measuring their total concentration is not sufficient to accurately evaluate their impact. Thus, a measurement and quantification of the metal and semimetal species present in water will provide a good indicator of its quality and, therefore, of the quality of the environment [13,14]. Additionally, these elements in the aquatic environment are susceptible to high temporal and spatial variabilities. This is why taking samples frequently becomes essential in order to provide a statistically valid estimate [4].

This study has been carried out in order to investigate the distribution, both spatially and temporally, of metals and potentially harmful substances in water, sediments and biota in order to assess and study their potential ecological risk in a very important industrialized area, such as Algeciras Bay (Southwestern Spanish coast) [15]. This aquatic ecosystem is surrounded by cities, industries and rivers with agricultural inputs, which influence the contamination of its waters, sediments and biota. Furthermore, it contains two of the most important European ports (Algeciras and Gibraltar ports) due to its high maritime traffic density, which makes it an area of great interest in comprehensive studies of contamination in aquatic environments. These significant sites show high values of anthropogenic stress index. These values have been described in a range of 90 to 150 for inner bay sites due to the high influence of human activities [16]. Few studies have evaluated this impact by the assessment of trace metal(loid) concentrations in the different biogeochemical phases. An integrated sediment assessment method classified the sediments from the Algeciras port, the Algeciras city and the northern part (with the major industries in the area) with a moderate degradation. High concentrations of Ni (50–82 mg/kg) and Cr (95–134 mg/kg), exceeding effects range low (ERL), were found [17] outstanding the mobile fraction of sediment and the correlation between them [18]. High Ni bioavailability was found in an area near a steel manufacturing plant where Ni is used in the manufacturing of alloys; the intermittent discharges of wastewater of the plant were not detected in spot analysis of water but Ni bioaccumulation was found in barnacle samples [18]. Ni and Co have been reported to be associated with PAHs in the oil spills occurring in this bay caused by the industrial plants and maritime activities, but also from various local activities [5]. A study of As concentrations in mussels from Algeciras over a 20-year sampling period showed that its source was not always associated with potential anthropogenic pressure [19]. The anthropogenic inputs of this highly toxic metalloid usually come from pesticides and mining activities. Dissolved As from tributaries can be incorporated by phytoplankton species and the biotransformation of inorganic As into organic compounds can be produced. This bioaccumulation has been significant in different sites of the Spanish Mediterranean coast for mussel and the western area of the Golf of Cadiz for sole [19,20]. Another study of the behavior of pollutants in Algeciras Bay showed that most of the contaminant plume stays close to the source with a strong gradient of metal concentrations [21]. These few preliminary studies regarding the elements studied in the present work (As, Ni, Co and Cr) show the interest and need to carry out a broad assessment of this singular ecosystem in Algeciras Bay. Due to their wide range of applications in industries or as pesticides in agriculture and their potential toxicity, a global assessment of these elements is proposed, containing studies of both their bioaccumulation and availability, depending on their speciation in different aquatic compartments (water, sediments and biota). 

Consequently, several potentially harmful elements (Ni, Cr, As and Co) have been measured in sediments, water and tissues of four different fish species: Streaked gurnard (*Trigloporus lastoviza* (Bonnaterre, 1788)), Senegalese sole (*Solea Senegalensis* (Kaup, 1858)), black scorpionfish (*Scorpaena porcus* (Linnaeus, 1758)) and white seabream (*Diplodus sargus sargus* (Linnaeus, 1758)) during four consecutive samplings. Results obtained were statistically analysed in order to study correlations among fractions and compartments, and they were compared with other ecosystems and guidelines in order to gather information about the evolution and interrelationship of the existing pollution among the different environmental compartments in areas anthropogenically influenced, and its possible toxic effects on organisms.

## 2. Materials and Methods

### 2.1. Description of the Study Area

**Figure 1:**
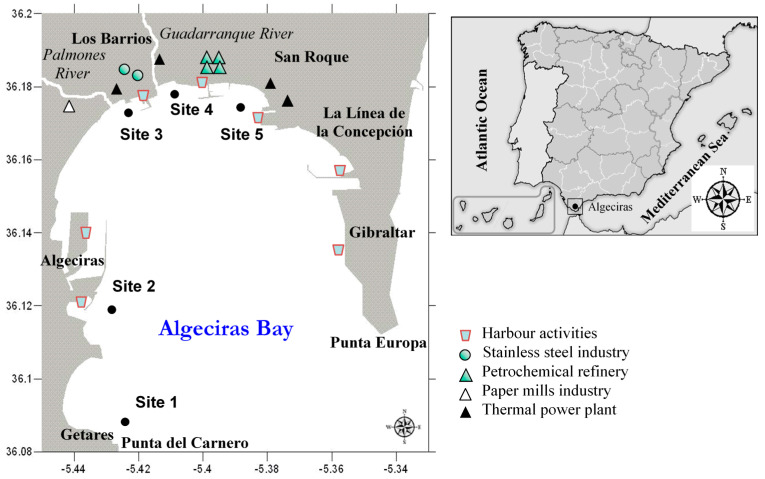
Map of Algeciras Bay showing the sampling sites and the principal anthropogenic activities of the area.

The Bay of Algeciras (Figure 1), 10–12 km long and 8–10 km wide, is a major industrialized area located in Cadiz, on the Southwestern coast of the Iberian Peninsula (Spain). It covers an area of approximately 75 km^2^ and is up to 400 m deep [22]. From several surrounding towns, such as Algeciras, San Roque, Los Barrios, Gibraltar and La Línea de la Concepción, a large amount of waste is discharged into the bay. During the study, untreated urban sewage was found in the bay. Additionally, two rivers flow into the bay: Guadarranque and Palmones. Another significant fact is the large number of industries scattered along the coast. These industries belong to different sectors, including: stainless steel manufacturing plants, hardware stores, oil industries, docks, shipyards and breakwaters [15,21,23]. Another impact factor is the intense activity from the two ports located in this bay: Algeciras and Gibraltar ports. The first one is ranked among the largest ports in Spain, while the latter is one of the Europe’s top ports for refueling [17]. Due to the proximity to the Strait of Gibraltar, the Bay of Algeciras has a high turnover, which is favoured by the currents formed by the confluence of the Atlantic Ocean and the Mediterranean Sea. These circumstances could assist the dispersion of some of the harmful elements in the water [15]. However, the increase in human activity in this area is leading to a gradual deterioration in the quality of the water, the sediments and the flora and fauna of the area, hence the need for it to be studied.

For that purpose, water, sediments and fish samples for speciation and total metal analysis were collected during four consecutive samplings (autumn 1 (sampling 1), spring 1 (sampling 2), autumn 2 (sampling 3) and spring 2 (sampling 4)). The samples were collected from: site 1, Getares beach, which was established as a control point due to the low human activity at the site; and four other sites considered to be highly polluted. The latter are located at sites: 2, Isla Verde, with a high presence of traffic, both road and maritime; 3, Palmones, which has the presence of the Acerinox steel manufacturing plant, a thermal power plant, a paper mill industry, urban influence and river activity; 4, Guadarraque, in the vicinity of which there are thermal power stations, Cepsa oil refineries and which is also influenced by urban areas and rivers; and finally 5, Puente Mayorga, with harbour activities and a thermal power plant (Figure 1). These four pollution hotspots were the ones with higher anthropogenic stress in the Bay, according to Guerra-García et al. [16], indicating high levels of human pressure [24]. Table 1 shows the geographical coordinates of each sampling site.

From each sampling site, one water sample and one sediment sample were collected during each sampling. As for fish, the number of specimens captured from each site is shown in Table A1 (in Appendix A).

### 2.2. Equipment

The equipment used to carry out the water samples extraction was a peristaltic pump (Masterflex 07571-05, poppet head 07518-02, Cole-Parmer Instrument Co., Vernon Hills, IL, USA). Samples were also filtered when necessary at the time of extraction, using a groundwater filter capsule (29705-92, Cole-Parmer Instrument Co., Vernon Hills, IL, USA) that was connected in-line to the Tygon tubes.

A portable electrochemical device, model Sension 156 (Hach Co., Loveland, CO, USA) was used to measure, also at the time of extraction, several parameters in the water samples, such as pH, temperature, salinity and dissolved oxygen (DO). In addition to the latter device, a TOC analyser (Shimadzu TOC Analyzer model 5050, Shimadzu, Columbia, MD, USA) was used to evaluate dissolved organic carbon. Organic matter content (% OM) in water and sediment samples was determined with a N 20/Hr muffle furnace (Nabertherm, Lilienthal, Germany). Digestions of water samples were carried out in Teflon reactors (PTFE, 100 mL, BRAND, 1305 38, Weirtheim, Germany).

Total and dissolved arsenic concentrations in water were analysed by hydride generation atomic absorption spectroscopy (HGAAS) using an Atomic Absorption Spectrometer coupled to a HG accessory Unicam VP90 (Thermo Elemental, Winsford, UK). A Metrohm 757 VA Computrace (Metrohm, Switzerland) with a hanging mercury drop electrode (HMDE) as working electrode was used to analyse the concentrations of Co and Ni present in the different fractions of water by differential pulse adsorptive cathodic voltammetry (DPAdCSV) and using the reagent dimethylglyoxime (DMG) at pH 9.5. The concentrations of Cr were analysed using a supporting electrolyte prepared with diethylenetriaminepentaacetic acid (DTPA) and sodium nitrate at pH 6.2 with acetate buffer, using the same DPAdCSV apparatus.

Sediments were shaken using an HS 501 D open air laboratory shaker platform (Ika, Labortechnik, Staufen, Germany). Sediment digestions were conducted using Siccatherm SICCA 250 W, 240 V, infrared lamps (Osram, Valencia, Spain). Fish tissues were freeze-dried employing a FreeZone Triad 7400030 equipment (Cole-Parmer, Vernon Hills, IL, USA) and microwave-assisted digestions were also carried out, using an Ethos 1600 microwave oven. (Milestone, Sorisole, Italy). Both sediment and fish samples were analysed using the X7 series sequential inductively coupled plasma scanning mass spectrometer. (Thermo Elemental, Winsford, UK).

Deionised water was ultrapurified by reverse osmosis with an Elix 3 (Milli-RO) system followed by ion exchange with an 18.2 MΩ cm (at 25 °C) Milli-Q50 deionised water system (Millipore, Burlington, MA, USA). A Basic 20 pH-meter with a 50_10T combined glass-Ag/AgCl electrode (Crison, Barcelona, Spain) was used in the laboratory to measure pH of the solutions. Solutions preparation and general sample handling were performed under an 870-FL vertical laminar flow cabinet (Cruma, Saint Boi de Llobregat, Barcelona, Spain).

### 2.3. Physical-Chemical Parameters Analysis

Using 0.45 µm nylon filters, the suspended solids (SS) in the water samples were gravimetrically quantified. Organic matter content (% OM) in water and sediment samples was estimated by measuring the loss of weight on ignition at 550 °C.

### 2.4. Water Collection and Analysis

Surface water samples were collected from a boat using a peristaltic pump which was coupled to flexible Tygon tubing and rigid Teflon tubing (6406-66). Unfiltered water samples were stored in low-density polyethylene bottles for subsequent analysis of metals and semimetals. For dissolved metal and semimetal analyses, water samples were filtered in situ and also collected into low-density polyethylene bottles. 

#### 2.4.1. Arsenic Speciation

Both filtered and non-filtered samples, used for arsenic speciation analysis, were acidified (at approximately pH 2) using 2 mL/L HCl (Suprapur grade). After that, they were stored for one week at room temperature and subsequently stored at −20 °C until analysis.

**Figure 2:**
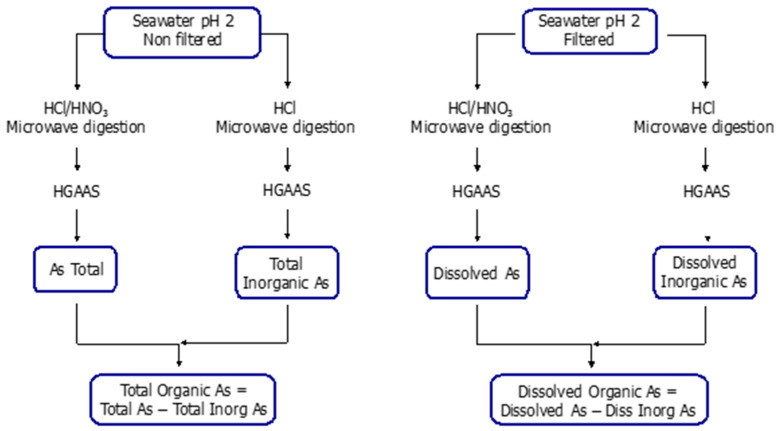
Scheme of chemical fractionation of As in water samples.

By operational discrimination between particulate and dissolved (organic and inorganic) arsenic species, arsenic speciation analyses were carried out by HGAAS [25]. As shown in Figure 2, a series of different acid digestions were performed. Using non-filtered and filtered water, total inorganic and dissolved inorganic arsenic fractions were analysed, respectively, after reduction in a microwave oven with HCl at a final concentration of 1% *v*/*v*. On the other hand, total and dissolved arsenic (both organic and inorganic) fractions were analysed after a microwave digestion with HCl at 1% *v*/*v* and HNO_3_ at 0.1% *v*/*v*.

The difference between total dissolved and inorganic dissolved As species provided the dissolved organic As concentrations, while particulate As concentrations were calculated from the difference between the total and the dissolved contents.

#### 2.4.2. Nickel and Cobalt Speciation

**Figure 3:**
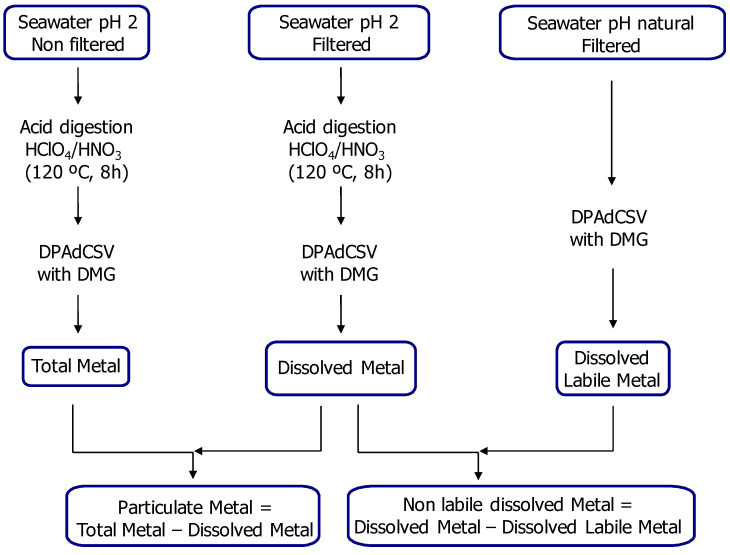
Scheme of chemical fractionation of Ni and Co in water samples.

In order to determine the total and dissolved metal contents, the filtered and non-filtered samples (500 mL) were acidified with 2 mL/L HNO_3_ (Suprapur grade) at approximately pH 2. After acidification, they were stored for one week at room temperature and then stored at −20 °C until analysis. One of the filtered samples (500 mL) was stored at −20 °C immediately after sampling at natural pH in order to evaluate the dissolved labile metal fraction according to fractionation schemes (Figure 3). An acid digestion was carried out on 45 mL of sample with 70% HClO_4_ (0.125 mL) and 65% HNO_3_ (0.2 mL) (Suprapur grade). The acid digestion was carried out in a Teflon reactor, at a temperature of 120 °C and for 8 h. It was then allowed to cool and diluted to 50 mL with Milli-Q water in a volumetric flask. After digestion, analyses of the total and dissolved Ni and Co fractions were determined by differential pulse adsorptive cathodic stripping voltammetry technique (DPAdCSV) and using dimethylglyoxime (DMG) at pH 9.5 as reagent, according to Metrohm (Metrohm, Application Bulletin, No. 231/2e) [26]. Using filtered water at natural pH, the dissolved labile fractions were analysed by DPAdCSV without pre-acid digestion. By calculating the difference between the dissolved metal fraction and the dissolved labile metal content, the non-labile dissolved metal concentration could be determined. To determine the particulate metal concentration, the difference between the total metal fraction and the dissolved metal fraction was calculated (Figure 3).

#### 2.4.3. Chromium Speciation

**Figure 4:**
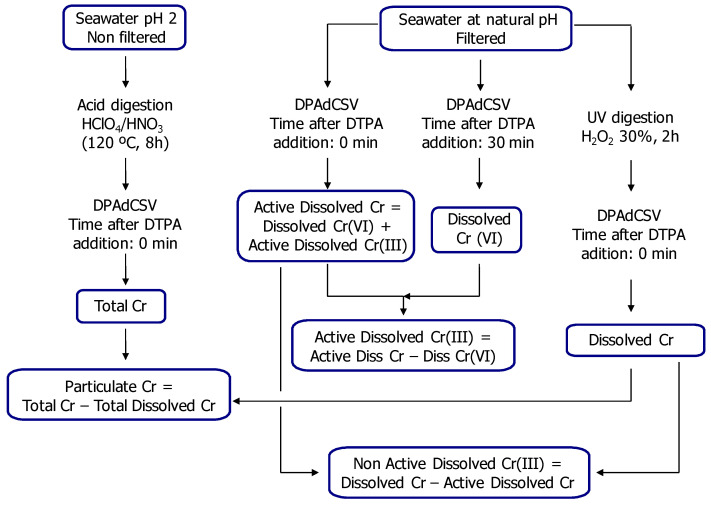
Scheme of chemical fractionation of Cr in water samples.

For the determination of total Cr, a previous digestion of the non-filtered water was performed. Afterwards, the analyses were carried out with DPAdCSV immediately after the addition of a supporting electrolyte prepared with diethylenetriaminepentaacetic acid (DTPA), sodium nitrate and acetate buffer at pH 6.2 (Metrohm, VA Application Note no. V-82; Application Bulletin, No 116/3e) [27,28]. In this way, inactivation of the Cr(III)- DTPA complex was avoided. The dissolved Cr content (including dissolved Cr(III) and Cr(IV)) was obtained after a 2 h digestion of the filtered water samples with 30% H_2_O_2_ (using 50 μL per 15 mL of water sample) and UV. The electrolyte was added and the analysis was carried out by DPAdCSV immediately afterwards. The contribution of active dissolved Cr(III) and dissolved Cr(VI) represents the active dissolved Cr content. The analysis of this fraction did not need a previous digestion and was carried out, as well, immediately after the addition of the electrolyte. Dissolved Cr(VI) concentration was determined by DPAdCSV 30 min after the addition of the electrolyte. The Cr(III)-DTPA complex was electrochemically inactivated after the waiting time. To determine the concentration of non-active dissolved Cr(III), the difference between the dissolved Cr and the active dissolved Cr contents was calculated. Additionally, the difference between active dissolved Cr and dissolved Cr(VI) provided the active dissolved Cr(III) content. The particulate Cr fraction was obtained by calculating the difference between the total Cr and the dissolved Cr contents. (Figure 4).

### 2.5. Analysis of Collected Sediments

Using an Eckman-Birdge dredge, surface sediment samples were collected from the seabed (about 2–20 cm deep). The samples were transported in polyethylene bags and stored at −20 °C until analysis. Subsequently, they were left to dry for one day at 105 °C for analysis. The samples were crushed (using an agate mortar) and, in order to obtain small particle size fractions (<0.063 mm) to work with, they were sieved using a stainless steel mesh. Finally, and until their sequential extraction, they were again stored in polyethylene bottles. 

The 3-step BCR extraction procedure described by Davidson et al. (1999) was used to proceed with sequential sample extractions [29]. First, 1 g of sediment samples were mechanically shaken with 40 mL of 0.11 mol/L acetic acid for 16 h at a speed of 150 rpm. Subsequent separation of the extractable fraction was performed by centrifugation. In the second step, the residue was mixed with 40 mL of 0.1 mol/L NH_2_OH-HCl (at pH 2, adjusted by addition of HNO_3_) for 16 h. Subsequently, the extract (the reducible fraction) was separated by centrifugation. The residue was then immersed twice in a water bath at 85 °C with 10 mL of 8.8 mol/L hydrogen peroxide (Suprapur grade), extracted using 50 mL 1.0 mol/L CH_3_COONH_4_ and finally separated by centrifugation, obtaining the oxidisable fraction. Using an IR lamp, the residual fraction was heated twice in a Teflon Petri dish with 5 mL HF 48% (Suprapur grade) until it was dried and then with two portions of 5 mL HNO_3_ 65% (Suprapur grade) until complete dryness. The residual fraction was leached by magnetic shaking and heating for 1 h, with 20 mL of 3.86 mol/L HCl.

The same procedure mentioned above for the residual fraction was carried out to obtain the total acid digestions of the sediments. The extracts obtained from the total acid digestions and from the sequential extractions were then adjusted to 50 and 25 mL, respectively. They were all stored in acid-washed polyethylene bottles at a temperature of 4 °C until analysis by inductively coupled plasma-mass spectrometry (ICP-MS). It should be noted that the respective blank samples were always prepared and taken into account. 

### 2.6. Analysis of Fish Collected

At the sampling sites mentioned above, fish were captured and dissected, obtaining different tissues, including portions of muscle, gills and liver. They were placed into liquid nitrogen at −80 °C to preserve the samples while they were being transported.

Samples of the above-mentioned tissues were freeze-dried and acid-digested using microwave heating. The liver samples (0.1–0.3 g) had a higher percentage of organic matter, so they were digested with 2 mL of 30% H_2_O_2_ (Suprapur grade) and 4 mL of 65% HNO_3_ (Suprapur grade). The muscle and gill samples (0.1–0.3 g), on the other hand, were digested using 7 mL of 65% HNO_3_. After digestion, a dilution to 25 mL with Milli-Q deionised water was carried out. Using ICP-MS, the digested tissue samples were analysed for metal content. Blanks for all metals were also analysed with the same procedure. 

### 2.7. Quality Assurance and Quality Control (QA/QC)

To store the collected water samples, LPDE bottles were cleaned with 3 mol/L HCl, later rinsed with Milli-Q deionised water and stored in polyethylene bags until required.

Using the same procedure as described above for each part of the speciation scheme, blanks were made with ultrapure water (Mili-Q). Acid-cleaned equipment was used for both the preparation of the solutions and the handling of the samples. Polyethylene gloves were also used and the procedures were performed under a laminar flow cabinet. Standard solutions for metal calibration curves were prepared in similar matrices as samples. Standards and blanks were also run between each 10 sets of samples for measurements quality control.

Limits of detection were calculated as (3 × s)/m, where s is the standard deviation of 10 blank measurements and m is the slope of the calibration curve. The results are shown in Table A2 (in Appendix A).

The quality of the analyses carried out on Ni, Cr and Co compositions was checked using the Slew-3 estuarine water as a reference material. Good results were obtained (94.5–101.5%, n = 3). On the other hand, the quality of the analyses performed on the As composition was checked using recovery tests with spiked samples. A good reliability of the analyses was demonstrated (98.5–102.2%, n = 3).

To check the quality of the sequential BCR extraction of Cr and Ni, standardised sediment reference material BCR-701 was used. Good results were obtained (91.6–120.3%, n = 4). Using standardised reference material from estuarine sediments NIST-SRM 1646a, the quality of the analyses of the total metal composition of the sediments was checked. Good reliability of the analyses was demonstrated (83.5–98.8%, n = 5).

Analysis of two biological reference materials certified by the National Research Council Canada was carried out in order to compare the accuracy of the methodology that had been carried out on tissue samples obtained from fish. The reference samples were as follows: NRCC DORM-2 (corresponding to dogfish muscle) and NRCC DOLT-3 (corresponding to dogfish liver). Satisfactory recoveries of 96.2–101.0%, n = 9 were obtained. 

### 2.8. Statistical Analyses

The STATISTICA 7 software package (STATSOFT 2004, Inc., Tulsa, OK, USA) was used to perform the different statistical analyses. Levene and Brown-Forsythe tests were used to measure the homogeneity of the data. On the other hand, the Shapiro-Wilk test (when n < 30) or the Kolmogorov-Smirnov test (when n > 30) was used to measure normality. Parametric tests were performed with the normally distributed and homogeneous data. Though, some data that were neither homogeneous nor normally distributed even when converted by various transformations (Log x, Log(1 + x), 1/x, 1/(1 + x), x^2^). Therefore, a series of non-parametric tests were carried out on these particular data. Significant differences among sampling dates and sites were studied using the parametric one-way ANOVA and Tukey tests or the non-parametric Kruskal-Wallis Rank tests and the multiple comparison tests (*p* < 0.05). Depending on the physical-chemical characteristics of the ecosystems as well as their water renovation patterns, metals and semimetals could be transferred from sediments to the water column, increasing their potential toxicity. This is why, in order to study the possible correlations of metal concentrations among the different compartments, the Pearson’s correlation coefficient (for homogeneous and normally distributed data) or the Spearman’s Rank correlation (*p* < 0.05) (for non-homogeneous nor normally distributed data) were calculated.

Thus, when studying the correlations among total metals in water and total metals in sediments, Pearson’s correlation test was performed. On the other hand, Spearman correlations were carried out in order to study the correlations of physicochemical parameters as well as the correlations of total metals and speciation data among the three compartments.

ANOVA tests were performed to study the significant differences of total metals in water among seasons and sampling dates, and total metals in sediments among seasons. On the other hand, Kruskall-Wallis and multiple comparison tests were carried out in order to study the significant differences of total metals in water among sampling sites, total metals in sediments among sampling dates and sampling sites, and speciation data in water, sediments and fish among seasons, sampling dates and sampling sites.

### 2.9. Pollution Indicators for the Assessment of Sediment Quality

Sediment pollution was assessed using several indices indicating the level of contamination. These indices were: the sediment geoaccumulation index (I_geo_), the enrichment factor (EF) and the contamination factor (CF). To provide background elemental concentrations for calculations, mean crustal abundance [30] and mean values for continental shales [31] are often used. In the present study, metal concentrations in continental shales (sedimentary rocks) have been selected as reference or background values for As (13 mg/kg), Ni (68 mg/kg), Co (19 mg/kg), Cr (90 mg/kg) and *Fe* (47,000 mg/kg) [31].

The enrichment factor (EF) was calculated using the following equation [32,33]:

(1) $$\mathrm{EF}=\frac{M_{x}\cdot Fe_{b}}{M_{b}\cdot Fe_{x}}$$

where *M_x_*, *M_b_*, *Fe_x_* and *Fe_b_* are the concentrations of studied metal or semimetal (*M*) and *Fe* in the sample and in the background reference, respectively. EF values were interpreted as: EF < 1 indicates no anthropogenic sources; and EF > 1 indicates presence of anthropogenic sources.

The contamination factor (CF) [31,34] was obtained by the ratio:

(2) $$\mathrm{CF}=\frac{\mathrm{Measured}\;\mathrm{concentration}\;\mathrm{of}\;\mathrm{the}\;\mathrm{metal}}{\mathrm{Background}\;\mathrm{level}\;\mathrm{of}\;\mathrm{the}\;\mathrm{metal}}$$

where CF ≥ 6 indicates very high level of pollution; CF = 3–6 represents significant or considerable level of pollution; CF = 1–3 indicates moderate pollution; and CF < 1 denotes low level of pollution. 

The geoaccumulation index (I_geo_) was calculated using the following expression [32,33,34]:

(3) $$I_{\mathrm{geo}}=\log_{2}\left(\frac{C_{n}}{1.5\cdot B_{n}}\right)$$

where *C_n_* is the measured concentration of the element *n* and *B_n_* is the background concentration for the average continental shale. The geoaccumulation index is associated with a qualitative scale of pollution status: I_geo_ < 0 indicates practically unpolluted status; I_geo_ = 0–1 denotes unpolluted to moderately polluted status; Ig_eo_ = 1–2 is moderately polluted status; I_geo_ = 2–3 represents moderately to strongly polluted status; I_geo_ = 3–4 is strongly polluted status; I_geo_ = 4–5 is strongly to extremely strong polluted status; and I_geo_ > 5 is extremely polluted status.

## 3. Results and Discussion

### 3.1. Physical-Chemical Parameters 

Table 2 shows the different parameters, both physical and chemical, found at each sampling site. In general, physical-chemical values were within the range of values for coastal systems. Temperature values ranged between 14.3–22.1 °C, with the lowest values in autumn 2 (sampling 3; mean: 15.2 °C). Algeciras water was slightly alkaline based on pH values recorded (7.0–8.6). Values of pH near 7 were probably due to river influence or discharges of sewage waters. Salinity values were found to be slightly lower in spring 2 (sampling 4), but they all were within the range of values established for coastal waters. Suspended solids (SS) values ranged from 0.014 to 0.040 g/L, exceeding at site 5 in autumn 1 (sampling 1) the mandatory value proposed by Andalusian Government (limit value for SS = 0.032 g/L) [35]. DOC content in water was quite remarkable in the same sampling and site (6.23 mg/L), exceeding the threshold value of 3 mg/L [35]. Organic matter in suspended solids was also higher in the same sampling and site. Some of the DO values were lower than the mandatory minimum value proposed by Andalusian Government (minimum value for DO = 70% sat) [35]. 

A high content of organic matter was observed in most of the sediments analysed, which could lead to a decrease in the availability of metals through complexation. Actually, average OM values in sediments were 13.6%, 2.6%, 8.4% and 4.0%, in each sampling respectively, surpassing in some cases the common values for organic matter in coastal sediment [36,37]. 

Dissolved oxygen (DO) in water, organic matter in suspended solids (OM in SS) and organic matter in sediments (OM) showed a seasonal tendency. According to the data, the natural occurrence of high biological activity in summer might have influenced these parameters. In fact, after summer season (in sampling 1 and 3) DO values were lower, while OM in suspended solids and in sediments showed higher contents, indicating the breakdown of organic matter due to the high biological activity. 

Temperature and DO values were compared to data obtained in the same ecosystem. Thus, temperature values were found to be similar while DO values obtained in this study were slightly lower than the ones found by Guerra-García et al. [16]. 

Spearman’s correlation showed a negative correlation between salinity and DO (*p* < 0.05; R_Spearman_= −0.74) since the presence of salts and dissolved solids usually decreases the solubility of gases in water. Dissolved organic carbon in water (DOC) showed a high correlation with SS (*p* < 0.05; R_Spearman_ = 0.73) and DO showed a negative correlation with the organic matter in sediments (*p* < 0.05; R_Spearman_ = −0.83), as expected. 

### 3.2. Metal and Semimetal Content in Water

#### 3.2.1. Total Metal and Semimetal Concentrations

The amount of total metals varied between the following ranges (Table 2): As: 0.5–0.8 μg/L, Ni: 0.1–0.6 μg/L, Co: 0.02–0.8 μg/L and Cr: 0.2–0.6 μg/L (As > Cr > Ni > Co). 

For As concentrations, few differences were found among the different samplings, with the highest As content being found at site 5. Comparing these data with the ones obtained in nearby ecosystems, As concentrations observed in water from Algeciras Bay were similar than those found in Cadiz Bay–a less industrialized area–where the values ranged from 0.16 to 1.96 µg/L. However, they were 5.2–11.6 times below the levels found in the Ría de Huelva–recognised as one of the most contaminated European estuaries–where As concentrations ranged from 2.6 to 9.3 µg/L [38,39]. In comparison with worldwide ecosystems, total As concentrations were 3.5 times below the levels found in Jinzhou Bay (China), where the average value was found to be 2.6 µg/L [40], 4 times below the ones found in the Al-Khobar coast (Saudi Arabian Gulf), where the values ranged from 0.1 to 3.5 µg/L [41] and 54 times below the ones reported in in Kuwait Bay [42], where the values ranged from <LOD to 43 µg/L.

Regarding Ni, it is very likely that the proximity of the stainless steel industry, the Palmones and Guadarranque estuaries and the oil refineries was the reason for the higher concentration found at sites 3 and 4 during autumn 2 (sampling 3) and site 4 during spring 2 (sampling 4). Although site 1 was originally selected as a control site, probably due to some kind of fuel spill it showed high concentration of total Ni during the first two samplings (autumn 1, spring 1). Later, from the third sampling on, a decrease in this high Ni concentration was observed, due to the good renovation of the waters, and the sanitation activities that were carried out in the area. Ni values were 21 times below those observed in the Ría de Huelva, where Ni concentration was between 2.1 and 13 µg/L [23]. In addition, these authors also studied the concentrations of Ni in the waters of Algeciras Bay during the first half of 2007, finding values of 0.4–1 µg/L, 1.6–4 times higher than those found in this study. Total Ni values were also 8–23 times lower than those found in San Francisco Bay (USA) and 4–15 times lower than the ones found in the Al-Khobar coast (Saudi Arabian Gulf) (0.8–13.7 µg/L [43] and 0.4–9 µg/L [41], respectively).

Co content was the lowest. However, high concentrations of this element were found at site 4 and, to a lesser extent, at site 3, probably due to the presence of a spill from the refinery. In comparison with other ecosystems, total Co in the Guadiana River Estuary ranged from 0.0326 to 0.194 µg/L [44], very similar to the values found in this study, except at site 4 in autumn (samplings 1 and 3). On the other hand, total Co concentrations were within those found in other ecosystems, such as the Hudson River Estuary (USA), San Francisco Bay (USA) and the Al-Khobar coast (Saudi Arabian Gulf) (0.028–3.231 µg/L [45]; 0.027–4.235 µg/L [45] and 0.1–0.79 µg/L [41], respectively).

The highest concentrations of Cr were found at sampling sites 2 and 4, as these areas are affected by urban, maritime traffic and industrial activities. Total Cr concentrations were also found to be 4–5.5 times lower than those in the Al-Khobar coast (Saudi Arabian Gulf) and in Liadong Bay (China) (0.8–3.3 µg/L [41] and 0.93–3.22 µg/L [46], respectively) and they were within the range (0.04–3.94 µg/L) found in Meiliang Bay (China) [47].

Significant differences among total metal and semimetal concentrations were investigated, studying sampling sites, sampling campaigns and seasons. Significance differences were observed for As between sites 3 and 5; and for Co between sites 1 and 4; although no significant differences were found among the samplings or seasons for any elements.

Slight negative correlations were found between As and Ni (R_Spearman_= −0.5422) and between Cr and Co (R_Spearman_= −0.5748), but no correlations were found among the physical-chemical parameters and any of the elements under study, indicating that the behaviour of total content was not associated with those parameters.

#### 3.2.2. Distribution of Dissolved and Particulate Metals and Semimetals

**Figure 5:**
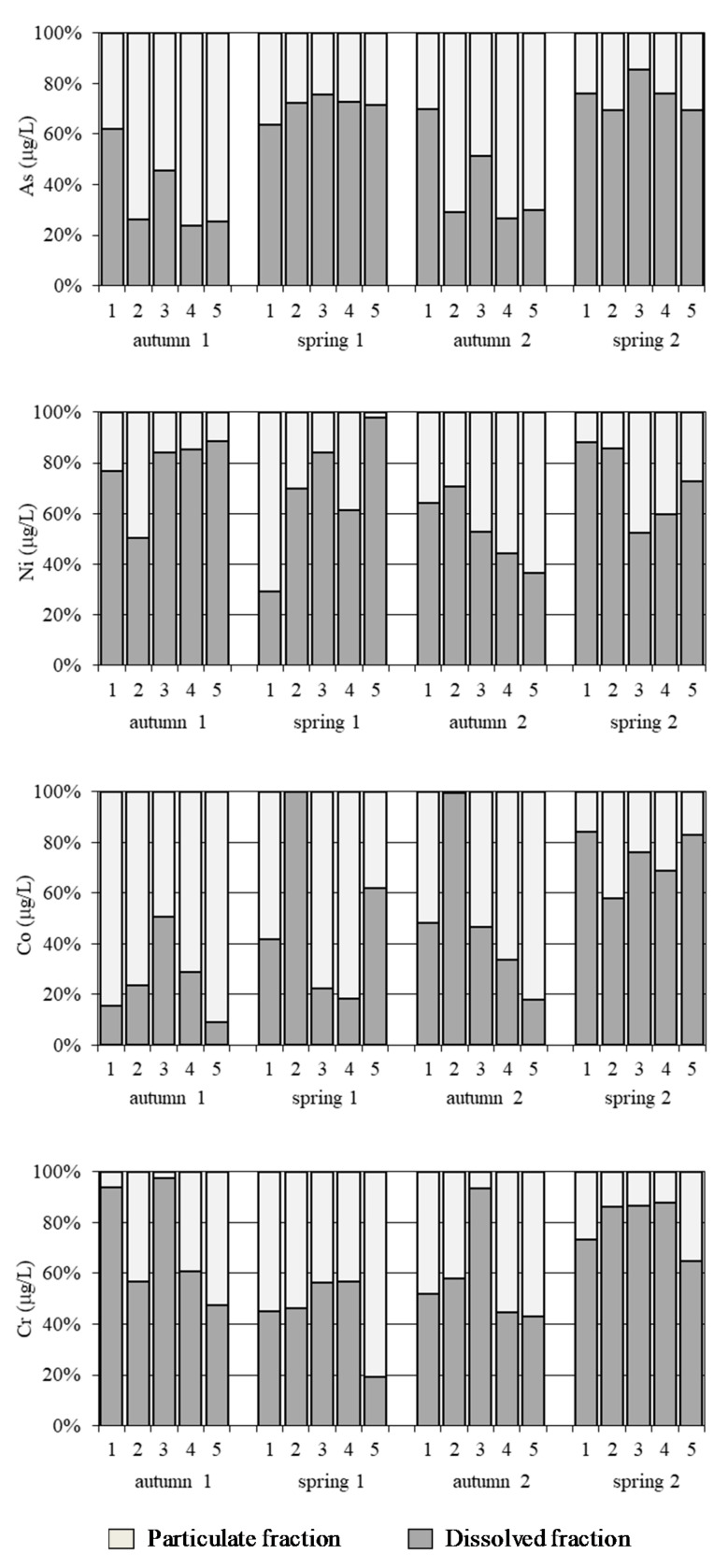
As, Ni, Co and Cr total concentrations and distribution of dissolved and particulate fractions in water samples at the 5 sites of Algeciras Bay.

Figure 5 shows the metal/semimetal distributions of dissolved and particulate fractions for each sampling and sampling site. It was generally observed that most metals and semimetals were mainly found in a high proportion in the dissolved fraction. A study of sediment transport model (for Zn) in Algeciras bay showed that metal dissolved concentrations from discharges of industrial and urban waste source decreased quickly, even inside the Bay. The metal stayed close to the source with a strong gradient of concentration, the weak transport inside the Bay was observed and the direct adsorption from dissolved phase on the sediment could take place [21].

The percentage of dissolved and particulate As varied depending on the season. Thus, during autumn (samplings 1 and 3), lower dissolved As content was observed (23.9–69.9%). Meanwhile, during spring (samplings 2 and 4), a higher percentage of dissolved As (63.8–85.5%) was obtained. Since suspended matter concentrations usually show seasonal variations due to river inputs by depending on pluviometry (rain episodes will enhance mean sedimentation rates) [21], the seasonal particulate As can be associated with inputs from the river with agricultural source. 

The majority of Ni content was found in its dissolved form (36.5–97.9%), with the exception of site 1 during the second sample collection, where a higher percentage (70%) of Ni was found in particulate form.

In terms of Co ratios, the samples varied greatly between high contents of particulate Co and others with a high percentage of dissolved metal. In the case of site 2 during the second and third samplings, for example, a dissolved percentage of 100% was found (Co percentages in dissolved form generally vary between 9.1 and 100%).

With regard to the proportions of Cr, a higher content of the particulate metal was found: at site 5, during the first three samplings (reaching 80% of particulate content during spring 1 (sampling 2)); at sites 1 and 2, during the second sampling (spring 1); and at site 4, during the third sampling (autumn 2). At the remaining sampling sites, the dissolved Cr content was found to be above 50% (51.8–97.4%).

As a result, the proportions of the dissolved fractions could be ordered from lowest to highest and, consequently, according to their potential bioavailability, as follows: Co < As < Cr < Ni. A higher dissolved metal concentration can provide a higher free metal ion and some complexes (defined by biotic ligand model) related with a higher metal bioaccumulation and toxicity [48]. 

**Figure 6:**
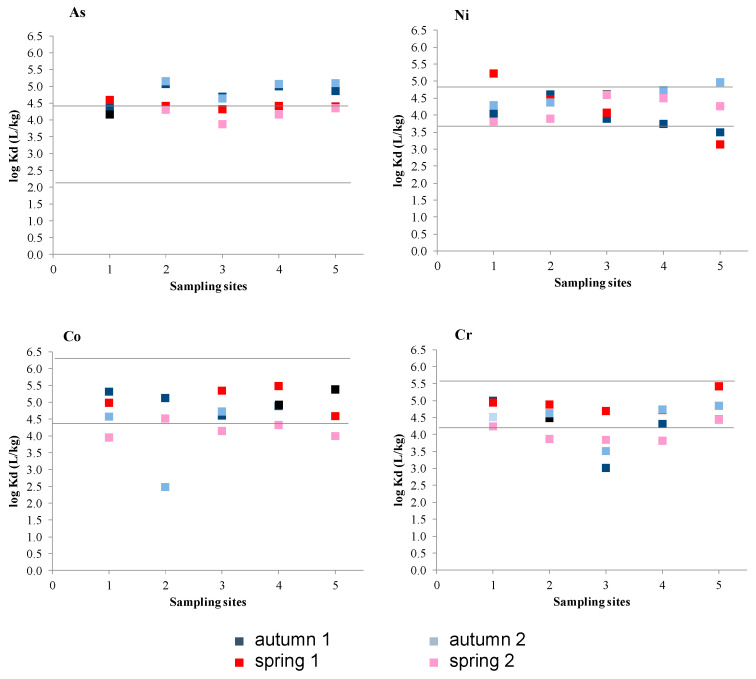
Values of log (K_d_ (L/kg)) for the four elements in each sampling site and comparison with other ecosystems (solid lines corresponds to minimum and maximum values found in other ecosystems).

The partitioning of these elements between particulate and dissolved phases has been calculated as the ratio of the element concentration in suspended particulate matter (*M*_particulate_) to that in the dissolved phase (*M*_dissolved_): K_d_ (L/kg) = *M*_particulate_/*M*_dissolved_. The four elements showed similar K_d_ values, indicating their similar behaviour in regards to their affinity for the dissolved phase. Figure 6 shows the Log K_d_ values obtained in all samplings and a comparison with values from different estuaries and bays [38,39,45,49,50,51,52,53,54]. The partition coefficients of As are higher than those found in other ecosystems, such as the Ria de Huelva (Spain), the Bay of Cadiz (Spain) and the Thames Estuary (UK) [38,39,51]. However, they are similar to those found in the Humber Estuary (UK) [52]. The K_d_ values obtained for Ni, Co and Cr are similar to those found in estuarine ecosystems and bays with high salinity [45,49,50,53,54].

Regarding possible correlations, little dependency of log K_d_ value on SS concentrations in Algeciras Bay was observed. Arsenic showed a slight positive correlation, likely due to adsorption reactions occurring during estuarine mixing, also above mentioned [21,55]. The K_d_ values of Co were nearly independent of SS concentrations, while inverse linear relationships were found for Ni and Cr, most likely due to the “particle concentration effect” which had been attributed to heterogeneity effects of particle size and composition [56]. According to Kruskall-Wallis and multiple comparison tests, dissolved As showed significant differences between autumn 1 and spring 1 (samplings 1 and 2), and autumn 1 and spring 2 (samplings 1 and 4). Further, significant differences between seasons were observed for dissolved As with higher concentrations in spring as above mentioned. Spearman’s correlations showed positive correlations between dissolved Cr and dissolved Co, but not with dissolved Ni. These results could be related to more different sources for Ni in the bay.

#### 3.2.3. Arsenic (As) Speciation Data 

**Figure 7:**
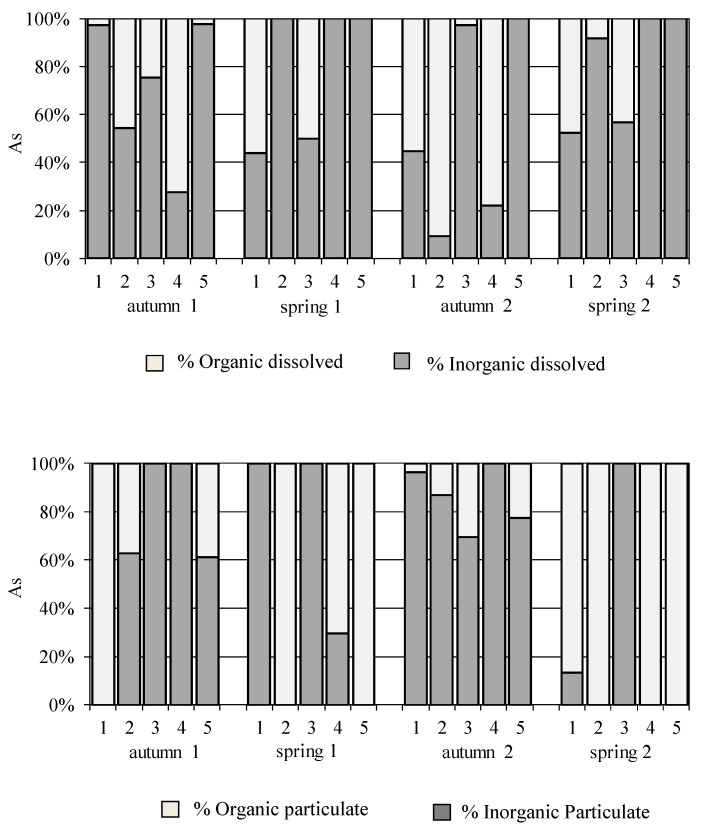
Distribution of inorganic and organic dissolved and particulate As in water samples of Algeciras Bay.

Figure 7 details the results obtained on the As speciation scheme applied during this investigation, which distinguishes between organic and inorganic As fractions, both particulate and dissolved. The inorganic fraction is considered the most harmful of them. In most of the samples collected, a higher proportion of inorganic As in dissolved form was detected (the most toxic fraction), mainly at site 5 (in all sampling), where almost 100% of the metal analysed was inorganic, at site 1 in the first sampling (autumn 1), site 2 and 4 in the second and fourth samplings (in spring) and at site 3 in the third sampling (autumn 2). For the particulate fraction, the percentage of inorganic As is highest, during all samplings at site 3, and also at site 1 in the second and third samplings and sites 2, 4 and 5 in autumn (the first and third samplings). The variation of the heavy metal concentrations in the particulate state is not only related to the properties of the heavy metals, but also is in relation to the current, particle size and the organic matter in the seawater. Bi et al. [57] pointed out that the organic matter in the particles and the content of fine particle grain were the key factors influencing the tidal cycles of particulate heavy metals. This can explain, in spring, high inorganic As in the dissolved phase inversely correlated with low inorganic As in the particulate phase, due to the lack of organic As forms related with the poor organic matter presents in suspended solids in water. 

#### 3.2.4. Cobalt (Co) and Nickel (Ni) Speciation Data 

**Figure 8:**
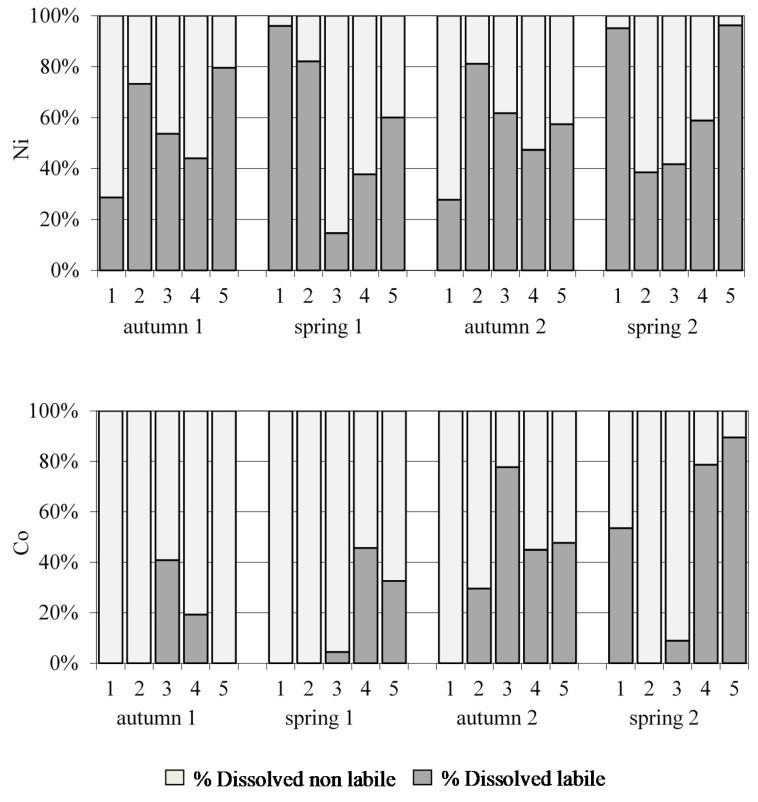
Distribution of dissolved labile and non-labile Ni and Co in water samples of Algeciras Bay.

From the speciation fractionation scheme, which is based on parameters such as pH, two metal fractions associated with the dissolved fraction were determined: the labile and non-labile fractions. Figure 8 shows the results, which indicate variations in labile Ni concentrations as a function of the sampling time. It was found that the non-labile fraction was higher at site 1 in autumn (samplings 1 and 3), site 3 in spring (samplings 2 and 4), site 2 in spring 2 (sampling 4) and site 4 in autumn 1, 2 and spring 1 (samplings 1, 2 and 3); while the labile fraction was predominant in the rest of samples, probably due to anthropogenic sources. With regard to Co speciation, the non-labile fraction was found to be generally higher than the labile fraction, suggesting that this metal could be less available to biota than Ni.

#### 3.2.5. Chromium (Cr) Speciation Data

**Figure 9:**
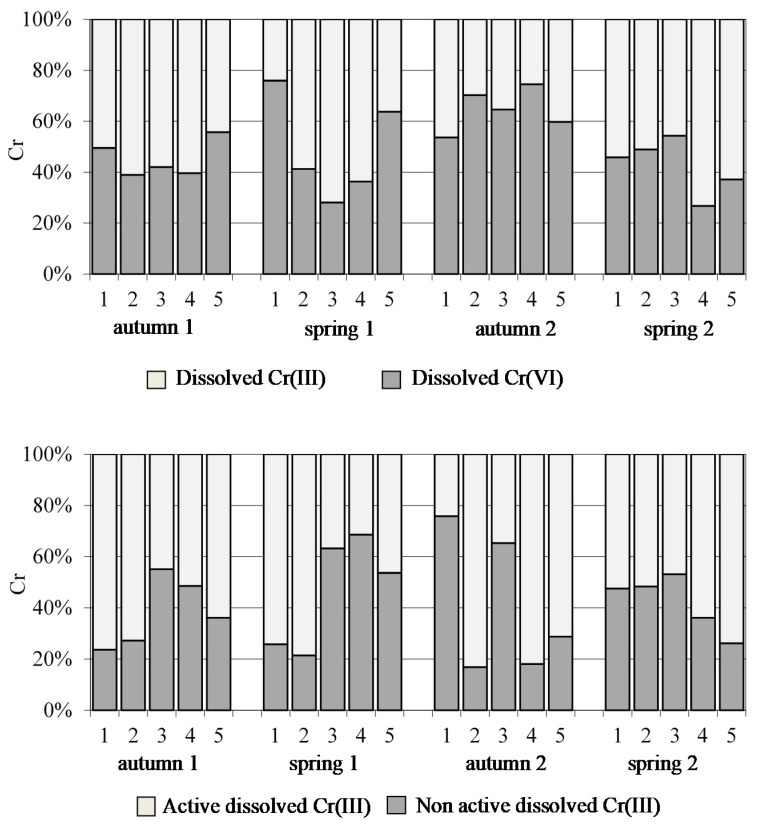
Distribution of dissolved Cr(III), dissolved Cr(VI), active dissolved Cr(III) and non-active dissolved Cr(III) in water samples of Algeciras Bay.

Figure 9 shows the speciation results obtained for Cr. From the speciation fractionation scheme based on pH, four different Cr fractions related to the dissolved portion of the metal were determined. These four fractions are: Cr(VI) in dissolved form (which is the most harmful of all), dissolved Cr(III), non-active dissolved Cr(III) and active dissolved Cr(III), which is the highest bioavailable form. Dissolved Cr(III) concentrations were higher than dissolved Cr(VI), except for all sites in autumn 2 (sampling 3), site 1 in spring 1 (sampling 2), site 3 in spring 2 (sampling 4) and site 5 in autumn 1 and spring 1 (samplings 1 and 2). In addition, at most sampling sites, the percentage of dissolved active Cr was found to be higher than its non-active form, suggesting that it could be highly bioavailable, with the exception of site 3 in all samplings, site 1 in autumn 2 (sampling 3), and sites 4 and 5 in spring 1 (sampling 2). On the other hand, the results also indicated that the non-active dissolved fraction of Cr(III) increased when dissolved Cr(III) was the dominant species over Cr(VI). The results generally indicated that Cr in water was found to be mainly in its most available form, although the least-toxic species was predominant.

### 3.3. Metal and Semimetal Content in Sediments

#### 3.3.1. Total Metal/Semimetal Concentrations

**Figure 10:**
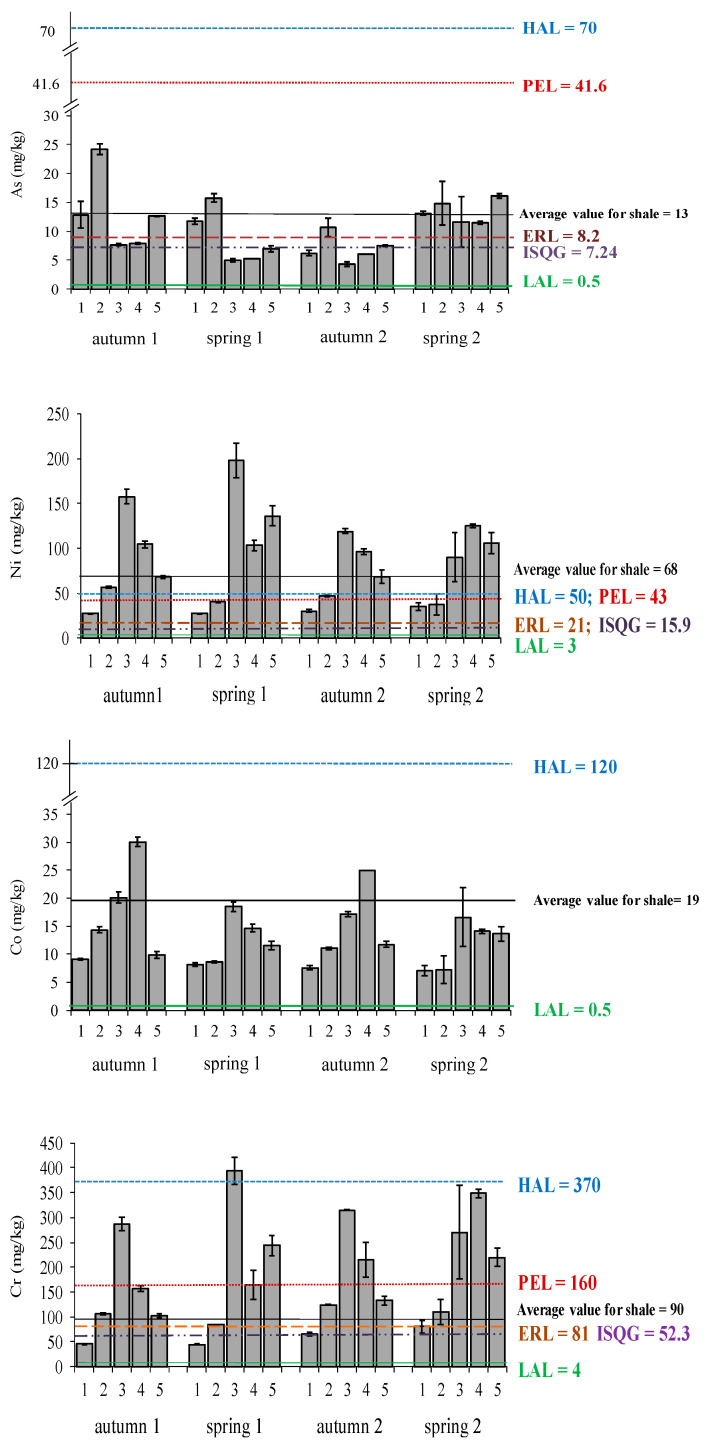
Total metal concentrations in sediments and comparison with quality guidelines (Low alert levels (LAL), effect range low (ERL), Interim Sediment Quality Guideline (ISQG), High Alert Level (HAL), Probable Effect Level (PEL) and average values for continental shale).

The content of metals and semimetals in sediments is shown in Figure 10. The values ranged as follows: As: 3.5–24.3 mg/kg, Ni: 8.2–198 mg/kg, Co: 3.6–30.1 mg/kg and Cr: 31.8–394.7 mg/kg. Thanks to the information collected, the different elements can be ordered from lowest to highest, according to their average content in the whole Bay, as follows: As < Co < Ni < Cr. The highest levels of As were found at site 2, which is close to the city of Algeciras, and at site 5, located close to the port. At site 3, where a stainless steel industry is located nearby, higher levels of both Cr and Ni are observed (these metals are extensively used in this kind of industries). At site 4, which is close to an oil refinery, the highest concentrations of Co in autumn and spring 2 (samplings 1, 3 and 4) were found, while in spring 2 (sampling 2) the highest values were given at site 3. No variations in the proportions of metal content were observed during the course of the different samplings.

In general, As concentrations observed in sediments from Algeciras Bay were similar than those found in Cadiz Bay, where the values ranged from 6.77 to 14.48 mg/kg and 1–2 times higher than the ones found in other ecosystems from China, such as the Jiaozhou Bay and Haizhou Bay [56,58,59]. However, they are 24–25 times below the levels found in the Ría de Huelva, where As concentrations ranged from 85.1 to 615.4 mg/kg [38,39]. 

Ni and Cr values in Algeciras Bay were above the concentrations found in other ecosystems, such as the Galician coast (affected by the oil spill from the tanker Prestige in November 2002), where Ni was found in the range 1.04–16.2 mg/kg [60], the Bohay Bay in China, where these metal concentrations ranged from 23.4 to 52.7 mg/kg for Ni and from 60.1 to 224.5 mg/kg for Cr [61] and other ecosystems, such as the Jiaozhou Bay and Haizhou Bay in China [56,58,59] or the Todos los Santos Bay in Brazil [62]. Morillo et al., 2007 found 3.7–5.0 times lower levels of Ni in Cadiz Bay (2.2–39.3 mg/kg) and up to 2 times lower in the same area of Algeciras Bay (16–100 mg/kg) [15]. Cr values found by Morillo et al., 2007 were 3.9–4 times lower in Cadiz Bay (8.14–42.0 mg/kg) and 1.6–2.7 times lower in the same area of Algeciras Bay (20–148 mg/kg) [15]. Regarding Co, concentrations found in Algeciras Bay were up to 15 times higher than those found in Galician coast, where Co values ranged from <LD to 2 mg/kg [60] and up to 1.9 times higher than those found in a semi-closed bay, the Longkou Bay in China [63].

According to statistical tests, Co, Cr and Ni showed significant differences (*p* < 0.05) among the different sampling sites. However, no significance temporal differences were found for any metal. The differences were found between sites 1 and 3 for Co, Cr and Ni; and between sites 2 and 3 for Cr and Ni. Co and Cr also showed significant differences between sites 1 and 4. It is very likely that because of the proximity of the steel plant, the oil refinery and the thermal power plants, higher levels of these metals were found at sites 3 and 4 than at site 1. 

Sediment pollutions indicators, such as Enrichment Factor (EF), Concentration Factor (CF) and Geoaccumulation Index (I_geo_) were calculated for all the samplings in order to assess the potential pollution of the sediments from Algeciras Bay (Table 3). These indicators have been extensively and recently used to assess metal pollution in aquatic ecosystems [41,64,65]. At all sites and for all metals, the EF values were >1 (except for As in site 3 and Ni and Co in site 1), indicating the presence of anthropogenic sources. The highest values were found for Cr in sites 2, 3, 4 and 5, and Ni in sites 3 and 4 because of the industrial influence; and for As in site 2 due to the port activity. 

The CF values indicated that most of the sites were considered as low or moderately polluted areas (with CF < 1 or 1 ≤ CF < 3, respectively), except for Cr in site 3, which was considered as considerably polluted (with 3 ≤ CF < 6). As for I_geo_, most of the sites were defined as unpolluted areas, except for Ni in site 3 and Cr in sites 4 and 5, which were considered unpolluted to moderately polluted areas, and Cr in site 3 which was considered as moderately polluted. These parameters showed that the sediments of the Bay of Algeciras are moderately polluted with Cr and Ni.

#### 3.3.2. Speciation of Metals/Semimetals

Sediments and suspended matter are important repositories for these elements, which may be present as discrete compounds, as ions held by cation-exchanging clays, bound to hydrated oxides of iron or manganese, or chelated by insoluble humic substances [66]. The distribution of metals in sediment (in percentage) following BCR procedure is shown in Table A3 and Table A4 (in Appendix A). Ni and Cr, in general, proved to be a high percentage of inert fraction in the sediments (70–90%) although the other fractions should be taken into account, mainly the exchangeable fraction (acid extractable) in sites 3, 4 and 5 for Ni. Co and As were mainly found in the acid exchangeable, oxidisable and reducible fractions, their mobilisation being easier. Based on the results, the potential availability of the elements present in the sediments can be ordered as follows: Cr < Ni < As < Co. Thus, although Cr showed the highest total content in sediments, it was observed to be mainly in the inert fraction. It is noteworthy that Co at site 4 recorded the highest total concentration levels and the lowest levels in the residual part. Thus, this metal can be exchangeable and potentially more available. 

Significant differences were observed between the different sample collection sites for all elements in the acid extractable and reducible fractions. However, only Co and Ni in the oxidisable and residual fractions, and As in the residual fraction, showed significant differences. This statistical study can indicate that the availability of each element may depend on the influence of the pollution hotspot.

### 3.4. Metal and Semimetal Content in Fish 

Metal content analysis was carried out on the different tissues (gills, liver and muscles) of the different fish species studied: *Solea senegalensis* (sole), *Trigloporus lastoviza* (streaked gurnard), *Scorpaena porcus* (black scorpionfish) and *Diplodus sargus sargus* (white seabream). Metal levels in gills usually reflect metal concentrations in the surrounding water; liver is an organ for storage and detoxification of metals, whereas muscle shows the potential pollution of fish as food. Gills are considered an important point of entry into the organism for essential and non-essential elements and are a useful tool for assessing metal bioavailability and accumulation in water. Metals reach the liver by gastrointestinal and bloodstream routes and their accumulation is related to the organ function (such as detoxification or antioxidation) and metallothionein synthesis. The metal concentration in muscle is commonly low with a fast decontamination rate [67]. Cr and Ni can induce formation of free radicals, which may also contribute to the development of hemolytic anemia [68]. Co is a cofactor for many enzymes and its toxicity to fish appears to be relatively low compared to the effects of other metal ions, particularly during short-term exposures. Cobalt toxicity causes heme oxidation and inhibition of inorganic calcium channels in fish gills [69]. Arsenic can accumulate in different tissues in various organic forms, with a low percentage in the more toxic inorganic form. Fish are described as good indicators of As toxicity in aquatic ecosystems, mainly planktivorous fish [70]. 

Table 4 and Table 5 show the average metal content found in each tissue. According to the observed data, the presence of the different elements in fish could be ordered as follows: Cr ≤ Ni < Co << As.

It could be seen that, in general, As accumulation occurred mainly in muscle and liver, rather than in gills. With the exception of site 5 during the first sampling (where there is an industrial plant nearby), and site 1 during spring 1 (sampling 2), As values did not vary among sites. At the two sites mentioned above, arsenic was found in a higher proportion in the liver: 367.73 mg/kg at site 5 and 225.33 mg/kg at site 1. It was also observed that arsenic levels in fish were higher in spring compared to those recorded in autumn. 

It was observed that Ni tended to accumulate in liver and gills. The highest values of Ni accumulated in gills were obtained during spring 2 (sampling 4) at site 4 (2.66 mg/kg). On the other hand, the highest levels of Ni accumulated in liver were found at site 1 during spring 1 (sampling 2; 13.85 mg/kg). There was no seasonal variation in the accumulated Ni levels. 

Co showed more tendency to accumulate in liver, then in gills and finally in muscle. The highest Co values found in gills were recorded at site 5 (6.70 mg/kg), while the highest levels in liver were found at both sites 4 (11.04 mg/kg) and 5 (10.29 mg/kg). The highest concentrations of Co were found in *Trigloporus lastoviza*, while the remaining elements (As, Cr and Ni) were found mostly in fish of the species *Solea Senegalensis*. 

Cr, with values ranging from 0.04 to 0.94 mg/kg, was found most in gill tissues, followed by muscle and finally liver.

The results obtained for sole tissues in Algeciras Bay were compared with other ecosystems. Thus, As mean levels found in this study were 1.3–4 times higher than those found in sole species from Cadiz Bay and Huelva Estuary, even though Huelva Estuary is considered a very polluted area [38,39]. Ni average concentrations are similar to those found in Cadiz Bay [71]. Mean concentrations of Co in gills and liver in fish from Algeciras Bay were 7 and 71 times higher, respectively, than those found in Cadiz Bay, although similar results in muscle as in this study have been reported [71]. Average values of Cr in gills and muscle in fish from Algeciras Bay were 2 and 20 times higher, respectively, than those found in Cadiz Bay, and similar results in liver have been reported [71]. Ferreira et al. (2008) found similar results of As in the liver and muscle of white seabream specimens [72].

Significance differences were found among the three tissues for all elements and among fish species for As and Co. In fact, As accumulation was significantly higher in *Solea Senegalensis*, while Co accumulation was significantly higher in *Trigloporus lastoviza*. No significant differences among species were found for Ni and Cr. Other significant differences among sites and samplings are shown in Table 6. 

The Metal Pollution Index (MPI) was also calculated in order to study the potential differences among sampling sites and tissues of fish in a joint way. This index represented the multiple metals present in the study area and is described as the geometric mean of trace metal concentration [73]:

MPI = (*C*_1_ × *C*_2_ × *C*_3_ …. × *C_n_*)^1/*n*^ (4)

where *C_n_* is the concentration of each metal *n*. 

**Figure 11:**
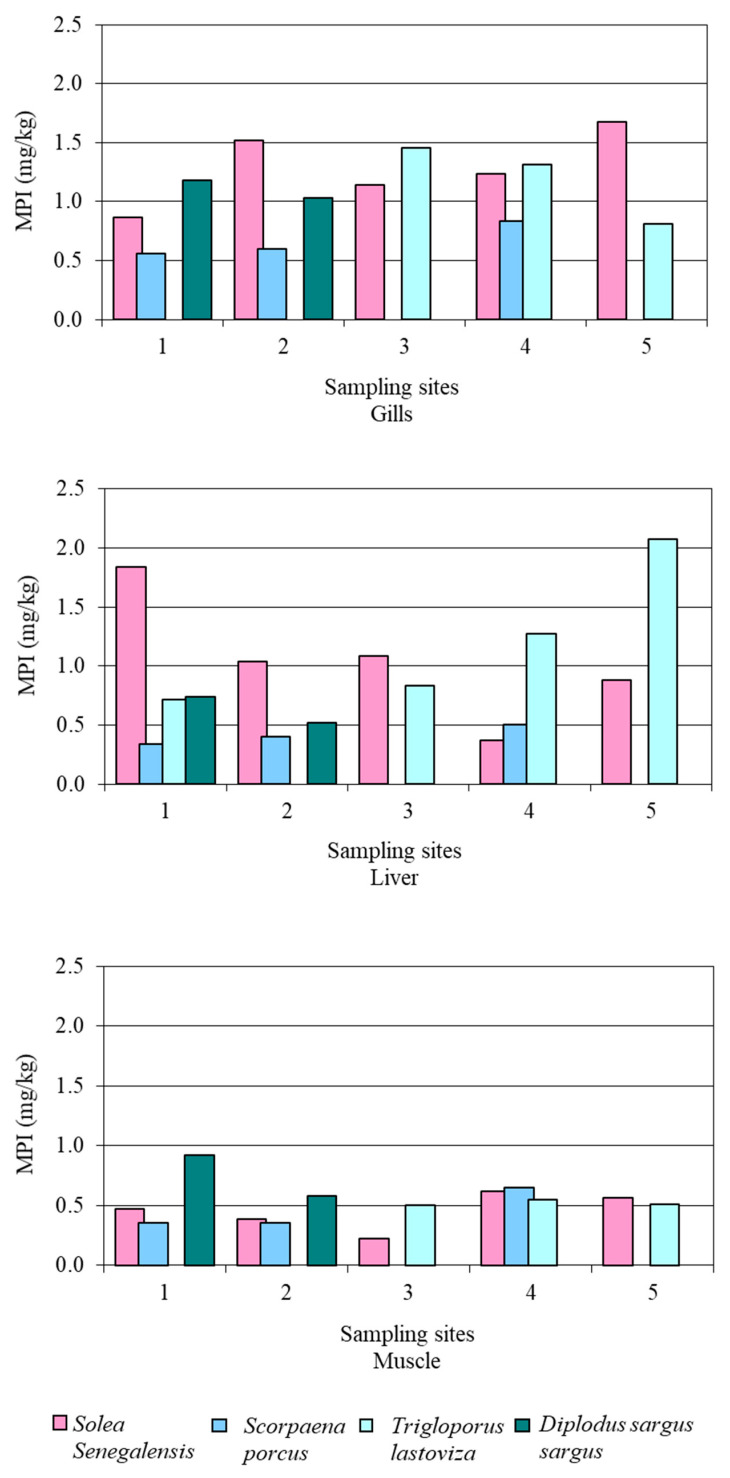
Metal pollution index for gills, liver and muscle of the four fish species collected at the five sampling sites.

As can be seen in Figure 11, it can be concluded that liver and gills are the target tissues, due to the fact that they accumulate metals more easily. Their content might allow differentiating between ecosystems with a different degree of contamination. On the other hand, bioaccumulation in muscle is not very useful to evaluate differences in contamination between ecosystems.

In order to study the translocation capacity of the metals and semimetals in water and sediments towards fish, the Bioconcentration Factor for water (BCF_w_) and for bottom sediment (BCF_BS_) was calculated as follows [74]:

BCF_w_ (mg L/μg kg) = *C*_fish_/*C*_water_ (5)

and

BCF_BS_ = *C*_fish_/*C*_sediments_ (6)

where *C*_fish_, *C*_water_ and *C*_sediments_ are the metals and semimetals concentrations in fish (mg/kg dry weight), dissolved fraction of water (μg/L) and total sediments (mg/kg dry weight), respectively. If BCF > 1, it indicates that the organism can accumulate these elements. BCF > 100 means that the bioaccumulation capacity of the organism is significant [74,75].

The BCF values are listed in Table A4 (in Appendix A) and the ranged values for water were as follows: 0.56–2116 for As; 0.01–130 for Ni; 0.15–1003 for Co; and 0.01–122 mg L/μg kg for Cr; while the values for sediments were: 0.02–30 for As; 0.00003–0.09 for Ni; 0.0002–0.94 for Co; and 0.00001–0.37 for Cr. BCF_w_ values in Table A4 were calculated with the dissolved metal content in water. In order to be able to compare the results from this study with others found in the literature, BCF_w_ values were also calculated with the total metal content in water. Thus, the ranges obtained were: 0.43–537 for As; 0.003–38 for Ni; 0.033–212 for Co; and 0.007–55 mg L/μg kg for Cr.

As expected, the accumulation capacity of these elements from water were much higher than those from sediments. However, the dissolved metal concentrations decrease quickly from the source and the effect of the metals is diluted. In both cases, As was the element with higher BCF, indicating that the studied species could accumulate As more easily than other metals. Most of the BCF calculated from water concentrations were higher than 1 and some of them were higher than 100, suggesting that these organisms have a high bioaccumulation ability for the studied elements. Generally, *Solea senegalensis* presented higher BCF for As, probably due to its benthic character, strongly associated with sediments from the bottom of the sea, where the labile and moderate labile fractions were significant.

Yuan et al. 2020 [74] studied the BCF_w_ in the dissolved fraction of water of bivalves from South China Sea. They found that all the BCF studied for Cd, Hg, Cu, Cr, Zn, Pb and As were higher than 100, suggesting that bivalves have a high bioaccumulation ability for heavy metals. According to these authors, the mean values of BCF for Cr and As ranged from 321 to 2502 mg L/μg kg and from 286 to 650 mg L/μg kg, indicating that the species of fish studied in the present research tended to accumulate less Cr and more As than bivalves.

Tang et al. 2020 [76] calculated the BCF_w_ values in dissolved fraction of water of aytid shrimp in tropical Taiwan and they found out that values for Cr ranged from 2 to 20 mg L/μg kg and values for As ranged from 0.04 to 0.12 mg L/μg kg, indicating that the species of fish studied in the present research tended to accumulate more Cr and As than this type of shrimps. 

Nędzarek et al. 2021 [77] calculated BCF_BS_ of Chinese mitten crabs from Germany. These values were calculated for muscle, hepatopancreas and gonads: 0.005, 0.013 and 0.170 for As; 0.005, 0.050 and 0.008 for Co and 0.017, 0.208 and 0.021 for Ni and 0.015, 0.009 and 0.004 for Cr, respectively. From this data, it can be concluded that the species of fish studied in the present research tended to accumulate more metals from sediments than this type of crab.

Saadati et al. 2020 [78] also studied the BCF_BS_ values for Ni in sentinel crabs from Persian Gulf. The obtained a mean value of 0.27 and 0.14 in soft and hard tissue, respectively. This data are higher than the ones obtained for the fish studied, indicating that this type of crabs tend to accumulate Ni from sediments more easily than the fish studied. 

Abdel Gawad, 2018 calculated the BCF_w_ in non-filtered water and BCF_BS_ values of mollusk gastropod from Egypt. BCF_w_ for Ni were 0.163 and 0.074 mg L/μg kg in shell and soft tissue, respectively; while for Co these values were 0.250 and 0.097 mg L/μg kg in shell and soft tissue, respectively [79]. These data indicate that the species of fish studied in the present research tended to accumulate more Ni and Co from water than these gastropods. Regarding BCF_BS_, the obtained values for Ni and Co were 0.335 in shell and 0.152 in soft tissue, and 0.390 in shell and 0.151 in soft tissue, respectively. This data are higher than the ones obtained for the fish studied, indicating that this type of gastropod tends to accumulate Ni and Co from sediments more easily than the fish studied.

Li et al., 2021 [80] studied the BCF_BS_ values of freshwater mussel Cristaria plicata from China, and they obtained values for As, Ni, Co and Cr in foot, gills, mantle and viscera mass. These results were as follows: 0.284, 0.599, 0.480 and 0.579 for As, 0.022, 0.068, 0.053 and 0.045 for Ni, 0.032, 0.124, 0.088 and 0.065 for Co and 0.068, 0.060, 0.096 and 0.081 for Cr. Comparing these results with the ones found in the present study, it can be concluded that As is more easily available from sediment in the fish studied than in the mussel Cristaria plicata, although the values of Ni, Co and Cr were within the range of the ones found in the present study.

Yang et al. [81] also studied the BCF_BS_ values of macrobenthic communities from China, and they obtained the following data: Malacostra showed values of 0.001–3.535 for As; 0.077–0.893 for Ni and 0.085–0.330 for Cr; while Bivalvia showed values of 1.110; 0.512 and 0.183 for As, Ni and Cr, respectively. These values showed that Malacostra and Bivalvia can accumulate As and Ni from sediments more easily than the fish studied, but they have the same tendency to accumulate Cr.

Sujitha et al. 2020 found BCFw in non-filtered water and BCFB_S_ values of different crabs from Mexico and they observed BCF_w_ values of 0.092; 0.213; 0.165 and 0.250 mg L/μg kg for As, Ni, Co and Cr, respectively [82]. These values were lower than the ones obtained for the studied fish, indicating that these crabs have lower ability to bioaccumulate these elements from water. Regarding BCF_BS_, the obtained values were 8.54; 0.53; 1.06 and 0.41 for As, Ni, Co and Cr, respectively, showing a greater ability to accumulate these elements from sediments.

### 3.5. Potential Hazardous Impacts of the Metals Studied in Algeciras Bay

Following a series of reference values (Table 7), the data obtained for the different metals/semimetals have been compared in order to quantify the harmful impact they are possibly causing in the environment. These reference levels are: background level [83], natural concentration [84], quality guidelines for protection of aquatic life proposed by several organizations [85] and imperative values of regional government for limited and non-limited waters [35]. Note that Algeciras Bay is classified as a non-limited area. The Ni and Cr contents in water were above the reference background values, although within natural concentrations. As concentrations were within the reference background values, so it did not represent a risk despite its high availability. It was observed that the concentrations of the elements present in all the samples were lower than those proposed by the Continuous Concentration (CCC) and EPA criteria of Maximum Concentration (CMC), which aim to prevent toxic effects that could harm the aquatic ecosystem. Although there are no reference CCC and CMC levels for Co concentrations and therefore it is not possible to determine its possible impact on the aquatic environment, much higher values were observed at site 4 during autumn (sampling 1 and 3) compared to values that can be found in natural seawater.

A comparison of the analysed elements in sediments was also performed with reference levels that determine their impact on biological activity (Figure 10). These reference values were established by the US National Oceanic and Atmospheric Administration (NOAA: National State and Trend program) [86], the Canadian Council of Ministers of Environment [87], the US Environmental Protection Agency [88] and Manheim and Commeau [89]. As mentioned above, these values are aimed at establishing adequate levels for the protection of the aquatic ecosystem. In addition, a comparison of the average values of the continental shale with the results obtained was carried out [31].

Arsenic levels exceeded ERL (Effect Range Low) and ISQG (Interim Sediment Quality Guideline) in many occasions. Ni levels also exceeded these values most of the time. Cr exceeded them practically in all cases during all samplings. Co is the element whose levels in the sediments were generally found to be within the natural range. HAL (High Alert Level) and PEL (Probable Effect Level) were extremely surpassed for Ni at sites 3 in samplings 1, 2 and 3 (autumn 1 and 2, spring 1), in sites 4 and 5 in samplings 1, 3 and 4 (autumn 1 and 2, spring 2), and at site 2 in autumn 1 (sampling 1). Regarding Cr, PEL level was surpassed at site 3 in all samplings, site 4 in spring 1–2 and autumn 2 (samplings 2, 4 and 3), and at site 5 in spring (samplings 2 and 4). HAL was exceeded at site 3 in spring 1 (sampling 2) (Figure 10).

Choueri et al. developed a site-specific sediment quality guidance for the Bay of Cadiz, which was more restricted than the national and international guidelines [90]. These guidelines included the metals Cd, Co, Cu, Ni, Pb, V and Zn. According to these authors, sediments were considered not polluted when Co ≤ 6.8 mg/kg and Ni ≤ 8.9 mg/kg; moderately polluted when 6.8 < Co < 14 mg/kg and 8.9 < Ni < 42.3 mg/kg and highly contaminated when Co ≥ 14 mg/kg and Ni ≥ 42.3 mg/kg. The values obtained for these metals in Algeciras Bay indicated that sites 2, 3, 4 and 5 during autumn (samplings 1 and 3), site 3 in spring 1 (sampling 2), site 4 in spring 2 (sampling 4) and site 5 in spring 1 and autumn 2 (samplings 2 and 3) were considered highly polluted regarding Ni levels, while the rest of sites were considered moderately polluted, except site 1 in autumn 2 (sampling 3), which was considered not polluted. Regarding Co levels, sites 2 in autumn 1 (sampling 1), site 3 in autumn 1–2 and spring 1 (samplings 1, 3 and 2), and site 4 in autumn 1–2 and spring 2 (sampling 1, 3 and 4) were considered highly contaminated, while the rest of sites were considered moderately contaminated, except site 1 in autumn 2 and spring 2 (sampling 3 and 4) and site 2 in spring 2 (sampling 4), which were considered not polluted.

On the other hand, according to OSPAR commission, the Marine Chemistry Working Group could not recommend guidelines for trace metals in fish, due to the limited dataset [91]. However, The Commission Regulation (EC) No 1881/2006 (and subsequent additions and amendments) sets maximum concentrations for contaminants in foodstuffs to protect public health, in order to ensure that contaminant concentrations are toxicologically acceptable. This regulation includes maximum levels for Pb, Hg and Cd in bivalve molluscs and fish muscle on a wet weight basis, but it does not include maximum levels for As, Ni, Co and Cr. Therefore, a comparison of these results and guidelines was not possible to achieve. 

Due to the fact that the elements studied are prone to accumulate in the sediments (especially Ni, Cr and As) and also thanks to the currents typical of this area that carry out the continuous renewal of these waters, it was concluded that, despite the high availability of the elements in the water samples, the dissolved concentrations do not represent a risk to the ecosystem. However, these elements often surpassed the guidelines in sediments, especially at sites 3 and 4, areas where As and Ni were found to be in the most available fractions. Thus, sediments in Algeciras Bay could be considered contaminated at sites 3 and 4, which were the areas influenced by industrial effluents.

### 3.6. Correlations among Water, Sediment and Fish

Correlations between trace elements in each compartments depend not only on internal processes which occur permanently in the aquatic environment, but also on the effect that human activities have on the partitioning and the way in which the elements behave in the environment [92]. In order to study the correlations among the three studied compartments, Spearman’s Rank correlation tests (*p* < 0.05) were carried out. Since significance differences between species were found for As and Co (Co concentrations in fish were higher in *Trigloporus lastoviza*, while the highest As concentrations were found in *Solea Senegalensis*), the dataset was treated accordingly to this information. Therefore, in order to study correlations between water/sediments and fish for these two metals, the species significantly different was studied separately from the rest. 

Total Co content in water highly correlated with total Co in sediments (R_Spearman_ = 0.75), Co content in the acid exchangeable and reducible phases (R_Spearman_ = 0.90 and 0.79, respectively). Additionally, total Co content in sediments also correlated with the acid exchangeable and reducible phases (R_Spearman_ = 0.89 and 0.87, respectively), indicating the strong association of this metal with sediments, especially with the most available fractions, as expected from the results found in the course of this research.

Moreover, total Ni content in sediments highly correlated with the oxidisable fraction (R_Spearman_ = 0.76), while Cr total content in sediments tightly correlated with the reducible, oxidisable and residual fraction (R_Spearman_ = 0.81; 0.79 and 0.77, respectively). This information was in agreement with the results obtained in this study, indicating that these two metals were mainly associated with the least available fractions of the sediment, being very difficult to mobilize them unless very extreme conditions exist.

No significant correlations were found between fish and water or fish and sediments, even though high metal contents were found in some fish tissues compared with other ecosystems, indicating that the industrial, maritime and urban activities in Algeciras Bay are affecting the biota of this ecosystem. 

## 4. Conclusions

During the course of this research, the levels of different elements present in sediment, water and fish tissues from Algeciras Bay were determined. In the sediments, Cr concentrations were the highest, followed by Ni, Co and finally As. In water, As concentrations were the highest, followed by Cr, Ni and finally Co. The different concentrations obtained were compared with reference values to determine the quality of the ecosystem. In many cases these values exceeded the reference levels indicated for sediments. On the other hand, speciation analyses of water and sediment samples were carried out to determine the potential bioavailability of the different elements, with Co and As being the most available in sediments and Cr and Ni in water. The possible impact of the elements on aquatic life was also determined by analysing the presence of the elements in different tissues (gills, muscle and liver) of four different fish species. The species analysed during the study were: *Scorpaena porcus*, *Diplodus sargus sargus*, *Solea senegalensis* and *Trigloporus lastoviza*. The concentrations of the elements found in the fish can be ordered as follows: Cr ≤ Ni < Co << As. While the highest levels of Cr and Ni were found in gills, Co and As concentrations were highest in liver. *Solea senegalensis* was the most affected species by As, due to its benthic habitat and to the labile As content in the sediments. 

In conclusion, although the levels of the elements analysed in water of Algeciras Bay were not of great concern and did not represent high contamination, the waste water and pollution from industrial activities, the maritime activities of the port and the urban centre of Algeciras itself could represent a risk to the sediments and aquatic life in the environment. Pollution indicators showed a moderate contamination of Ni, Cr and As, which should be taken into consideration.

## Figures and Tables

**Table 1:** Geographical coordinates of the sampling sites.

Sampling Sites	North	West
1 (Getares beach)	36°05′28.31″	5°26′10.93″
2 (Isla Verde)	36°07′08.43″	5°25′37.60″
3 (Palmones)	36°10′19.51″	5°25′27.14″
4 (Guadarranque)	36°10′32.21″	5°24′27.47″
5 (Puente Mayorga)	36°10′32.23″	5°23′23.24″

**Table 2:** Characterisation of the Algeciras Bay ecosystem.

Sampling	Sites	T (°C)	pH	Salinity (‰)	DO * (%sat, mg/L)	SS * (g/L)	OM in SS * (%)	DOC * (mg/L)	OM in S * (%)	Water Samples (μg/L)			Sediment Samples (mg/kg)				
										As	Ni	Co	Cr	As	Ni	Co	Cr
**Autumn 1** **(1st)**	1	21.2	8.34	35.6	47.7/4.0	0.027	32.9	1.72	16.1	0.625	0.378	0.022	0.200	12.8	27.2	9.1	45.5
	2	22.1	8.27	35.5	48.3/4.1	0.024	17.2	1.52	17.8	0.684	0.285	0.035	0.422	24.3	56.6	14.3	106.4
	3	20.1	8.25	37.2	43.7/4.0	0.024	26.5	1.39	12.3	0.556	0.294	0.099	0.310	7.7	157.8	20.1	286.8
	4	21.3	8.24	35.2	45.2/4.0	0.032	25.7	1.53	11.6	0.665	0.271	0.559	0.493	7.9	104.4	30.1	156.1
	5	21.0	7.03	34.4	44.5/4.1	0.040	35.0	6.23	10.2	0.702	0.233	0.047	0.344	12.6	67.9	9.8	102.3
**Spring 1** **(2nd)**	1	18.3	8.42	34.8	85.8/6.6	0.014	12.8	0.41	2.6	0.740	0.367	0.032	0.304	11.7	27.3	8.1	44.7
	2	19.7	8.42	33.9	90.4/7.0	0.015	18.2	0.59	5.3	0.627	0.232	0.046	0.555	15.8	40.1	8.7	85.5
	3	20.2	8.56	32.4	88.8/6.9	0.016	13.4	0.67	1.8	0.550	0.276	0.122	0.478	4.9	198.1	18.5	394.7
	4	20.9	8.51	33.8	89.6/6.9	0.015	20.2	0.72	1.7	0.494	0.240	0.143	0.520	5.3	103.5	14.6	164.0
	5	18.9	8.58	33.5	88.5/6.9	0.016	19.0	0.56	1.5	0.795	0.149	0.107	0.495	7.0	136.2	11.5	243.6
**Autumn 2** **(3rd)**	1	14.9	7.98	32.9	77.5/5.7	0.029	19.4	1.74	4.1	0.556	0.284	0.044	0.408	6.2	30.3	7.6	64.8
	2	15.9	8.03	32.8	75.1/5.4	0.017	12.6	1.03	7.6	0.616	0.137	0.034	0.426	10.7	47.2	11.0	124.2
	3	15.4	8.06	32.2	67.7/4.7	0.022	17.9	0.66	6.8	0.496	0.484	0.153	0.287	4.3	119.2	17.2	314.7
	4	15.3	8.04	31.2	73.5/5.3	0.024	21.4	1.67	7.6	0.600	0.536	0.752	0.538	6.0	96.1	24.9	214.3
	5	14.3	8.07	32.4	66.8/4.6	0.019	17.8	1.52	15.7	0.707	0.197	0.064	0.477	7.5	68.0	11.7	132.8
**Spring 2** **(4th)**	1	18.2	7.11	30.1	88.0/7.5	0.021	12.8	0.92	3.1	0.734	0.189	0.030	0.324	13.1	35.6	7.1	79.9
	2	18.3	6.96	29.3	83.5/7.2	0.022	16.1	2.51	5.7	0.668	0.245	0.029	0.392	14.9	37.0	7.2	110.4
	3	18.2	7.58	30.3	95.5/7.9	0.023	14.6	0.41	2.4	0.565	0.294	0.064	0.383	11.6	90.3	16.6	269.9
	4	18.6	7.37	29.5	88.4/7.5	0.022	12.6	1.05	4.5	0.561	0.574	0.053	0.395	11.5	125.3	14.1	348.7
	5	18.5	7.42	29.7	94.0/7.7	0.020	7.1	1.14	4.4	0.779	0.146	0.037	0.302	16.1	106.2	13.6	219.7

* DO: dissolved oxygen; SS: suspended solids; OM in SS: organic matter in suspended solids; DOC: dissolved organic carbon in water; OM in S: organic matter in sediments.

**Table 3:** Sediment pollution indicators in sediments from Algeciras Bay.

Sediment Pollution Indicator	Sites	As	Ni	Co	Cr
EF	1	1.67	0.70	0.84	1.16
	2	2.75	1.44	1.27	2.60
	3	0.60	2.63	1.25	4.52
	4	1.12	2.72	2.13	4.97
	5	1.67	2.19	1.20	3.62
CF	1	0.64	0.27	0.33	0.45
	2	1.11	0.58	0.51	1.05
	3	0.43	1.89	0.90	3.24
	4	0.55	1.35	1.06	2.47
	5	0.81	1.06	0.58	1.75
I_geo_	1	−1.22	−2.48	−2.20	−1.74
	2	−0.44	−1.37	−1.55	−0.52
	3	−1.79	0.33	−0.74	1.11
	4	−1.43	−0.15	−0.50	0.72
	5	−0.89	−0.50	−1.37	0.23

**Table 4:** Average metal concentration in fish and standard deviation at each sampling site (mg/kg).

Sampling Site	Tissue	Mean Value ± s.d.			
		As	Ni	Co	Cr
1	Liver (n = 32)	33.09 ± 45.96	0.62 ± 2.42	0.66 ± 1.18	0.59 ± 2.93
	Gills (n = 40)	5.66 ± 3.86	0.52 ± 0.47	0.25 ± 0.28	0.74 ± 0.65
	Muscle (n = 40)	36.63 ± 21.88	0.17 ± 0.37	0.06 ± 0.11	0.27 ± 0.18
2	Liver (n = 8)	34.73 ± 56.02	0.19 ± 0.12	1.12 ± 1.26	0.09 ± 0.19
	Gills (n = 9)	12.73 ± 12.10	0.49 ± 0.41	0.36 ± 0.19	0.54 ± 0.72
	Muscle (n = 8)	47.66 ± 52.81	0.10 ± 0.13	0.08 ± 0.08	0.22 ± 0.12
3	Liver (n = 18)	21.56 ± 18.54	0.24 ± 0.21	1.88 ± 1.27	0.10 ± 0.18
	Gills (n = 18)	14.12 ± 10.72	0.57 ± 0.28	0.52 ± 0.23	0.94 ± 0.74
	Muscle (n = 21)	27.75 ± 29.41	0.05 ± 0.10	0.25 ± 0.74	0.14 ± 0.12
4	Liver (n = 11)	11.80 ± 8.95	0.42 ± 0.44	1.62 ± 3.13	0.04 ± 0.04
	Gills (n = 12)	7.30 ± 5.51	0.75 ± 0.71	0.61 ± 0.44	0.64 ± 0.55
	Muscle (n = 11)	27.28 ± 16.32	0.14 ± 0.18	0.12 ± 0.06	0.48 ± 0.53
5	Liver (n = 7)	82.88 ± 131.83	0.27 ± 0.23	1.90 ± 4.04	0.13 ± 0.08
	Gills (n = 8)	20.10 ± 25.24	0.35 ± 0.25	1.21 ± 2.25	0.56 ± 0.43
	Muscle (n = 7)	73.88 ± 49.87	0.14 ± 0.21	0.04 ± 0.04	0.32 ± 0.21

**Table 5:** Average metal concentration and standard deviation for each fish species (mg/kg).

Species	Tissue	Mean Value ± s.d.			
		As	Ni	Co	Cr
*Solea senegalensis* (n = 45)	Liver	46.13 ± 71.39	0.56 ± 2.16	0.39 ± 0.51	0.52 ± 2.62
	Gills	9.60 ± 13.13	0.60 ± 0.55	0.36 ± 0.96	0.89 ± 0.71
	Muscle	53.46 ± 34.16	0.11 ± 0.14	0.03 ± 0.02	0.26 ± 0.18
*Scorpaena porcus* (n = 11)	Liver	8.29 ± 5.48	0.20 ± 0.23	0.99 ± 1.09	0.03 ± 0.03
	Gills	4.20 ± 2.14	0.42 ± 0.29	0.38 ± 0.17	0.33 ± 0.26
	Muscle	15.34 ± 10.90	0.10 ± 0.14	0.11 ± 0.07	0.29 ± 0.17
*Trigloporus lastoviza* (n = 22)	Liver	20.81 ± 17.65	0.30 ± 0.31	3.22 ± 2.97	0.06 ± 0.07
	Gills	14.03 ± 9.46	0.49 ± 0.31	0.69 ± 0.38	0.69 ± 0.65
	Muscle	22.10 ± 16.89	0.06 ± 0.11	0.27 ± 0.74	0.28 ± 0.43
*Diplodus sargus sargus* (n = 5)	Liver	16.46 ± 6.21	0.32 ± 0.25	0.82 ± 0.69	0.06 ± 0.04
	Gills	8.89 ± 3.70	0.60 ± 0.45	0.58 ± 0.71	0.54 ± 0.19
	Muscle	18.64 ± 12.49	0.65 ± 0.95	0.18 ± 0.31	0.25 ± 0.17

**Table 6:** Significant differences among sites and samplings in fish from Algeciras Bay.

Tissue	Metals			
	As	Ni	Co	Cr
Gills	Sole–others spring 1–autumn 2 spring 1–spring 2	autumn 2–spring 2	Sole–others spring 1–spring 2 autumn 2–spring 2	autumn 2–others
Liver	Sole–others spring 1–spring 2		Sole–others autumn 2 –spring 2	autumn 1–autumn 2
Muscle		spring 1–spring 2 autumn 2–spring 2	Sole–others autumn 1–spring 2	sites 3–4 spring 1–autumn 2 spring 1–spring 2

**Table 7:** Background levels, natural concentration and guidelines levels of metals (μg/L) in water described in the literature.

Metal	Background Level (Förstner and Wittman. 1983)	Natural Concentration (Azcue. 1993)	NOAA-EPA (USEPA, 2016)		Quality of Water (BOJA 27 14/2/1997) ^c^	
			CMC ^a^	CCC ^b^	Classification of Waters	Imperative Values
As	2.1	1.3–2.5	69	36	Limited	50
					Non limited	25
Ni	0.2	0.02–0.7	74	8.2	Limited	50
					Non limited	25
Co	0.04	0.02	-	-	Limited	-
					Non limited	-
Cr	0.08 ^d^	0.09 ^d^–0.55 ^d^	10,300 ^e^ 1100 ^f^	50 ^f^	Limited	20 ^d^/6^f^
					Non limited	10 ^d^/4 ^f^

^a^ CMC: criteria maximum concentration (dissolved metal); ^b^ CCC: criteria continuous concentration (dissolved metal); ^c^ IV: imperative values (total metal) proposed by Andalusian Government (Spain); ^d^ Total Cr; ^e^ Cr(III); ^f^ Cr(VI).

## Data Availability

Data is contained within the article or appendices.

## Appendix A

**Table A1:** Biometric characteristics of fish found in Algeciaras Bay and Bioconcentration factors from water and bottom sediments (n.d.: non detected; L: liver; M: muscle; G: gills).n.d.: non detected; L: liver; M: muscle; G: gills.

Samplings	Sites	Sample	Species	Tissue	Length (cm)	Weight (g)	BFC_W_				BCF_BS_			
							As	Ni	Co	Cr	As	Ni	Co	Cr
Autumn 1	1	1	*Diplodus sargus sargus*	L	143	21	52	0.2	30.4	0.4	1.6	0.002	0.012	0.002
Autumn 1	1	1	*Diplodus sargus sargus*	G	143	21	11	4.6	532.4	4.5	0.3	0.049	0.202	0.019
Autumn 1	1	1	*Diplodus sargus sargus*	M	143	21	12	8.0	210.9	2.5	0.4	0.086	0.080	0.011
Autumn 1	1	1	*Solea senegalensis*	L	236	29	36	0.4	53.9	1.5	1.1	0.004	0.020	0.006
Autumn 1	1	1	*Solea senegalensis*	M	236	29	72	n.d.	20.9	1.4	2.2	n.d.	0.008	0.006
Autumn 1	1	1	*Solea senegalensis*	G	236	29	11	1.6	79.9	7.9	0.3	0.017	0.030	0.033
Autumn 1	1	2	*Solea senegalensis*	L	115	23	33	0.2	39.4	1.2	1.0	0.002	0.015	0.005
Autumn 1	1	2	*Solea senegalensis*	M	115	23	38	0.4	10.1	2.7	1.2	0.005	0.004	0.011
Autumn 1	1	2	*Solea senegalensis*	G	115	23	11	0.5	52.4	5.2	0.3	0.006	0.020	0.022
Autumn 1	1	3	*Solea senegalensis*	L	225	28	53	0.1	28.4	0.6	1.6	0.002	0.011	0.003
Autumn 1	1	3	*Solea senegalensis*	G	225	28	14	0.3	31.6	4.5	0.4	0.004	0.012	0.019
Autumn 1	1	3	*Solea senegalensis*	M	225	28	52	n.d.	15.1	1.1	1.6	n.d.	0.006	0.005
Autumn 1	3	1	*Trigloporus lastoviza*	M	355	32	18	0.4	2.3	1.7	0.6	0.001	0.006	0.002
Autumn 1	3	1	*Trigloporus lastoviza*	L	355	32	10	0.5	20.2	0.1	0.3	0.001	0.050	0.000
Autumn 1	5	2	*Trigloporus lastoviza*	M	76	20	103	0.4	33.3	2.1	1.5	0.001	0.014	0.003
Autumn 1	5	2	*Trigloporus lastoviza*	G	76	20	85	0.5	48.5	1.2	1.2	0.002	0.020	0.002
Autumn 1	5	2	*Trigloporus lastoviza*	L	76	20	112	2.6	184.3	1.4	1.6	0.008	0.075	0.002
Autumn 1	5	4	*Solea senegalensis*	M	359	33	759	1.3	12.5	3.8	10.7	0.004	0.005	0.006
Autumn 1	5	4	*Solea senegalensis*	L	359	33	2116	2.3	46.8	1.1	29.9	0.007	0.019	0.002
Autumn 1	5	4	*Solea senegalensis*	G	359	33	150	3.5	61.8	7.8	2.1	0.011	0.025	0.012
Spring 1	1	5	*Solea senegalensis*	M	135	24	88	1.5	3.8	1.9	3.5	0.006	0.006	0.006
Spring 1	1	5	*Solea senegalensis*	L	135	24	33	1.6	20.3	n.d.	1.3	0.006	0.033	n.d.
Spring 1	1	5	*Solea senegalensis*	G	135	24	11	4.0	17.1	2.6	0.4	0.016	0.028	0.008
Spring 1	1	6	*Solea senegalensis*	M	136	24	99	n.d.	1.3	0.5	4.0	n.d.	0.002	0.002
Spring 1	1	6	*Solea senegalensis*	G	136	24	16	2.9	12.1	7.3	0.7	0.011	0.020	0.022
Spring 1	1	7	*Solea senegalensis*	M	114	21	56	1.5	3.0	1.8	2.2	0.006	0.005	0.006
Spring 1	1	7	*Solea senegalensis*	G	114	21	15	15.8	14.4	7.5	0.6	0.062	0.024	0.023
Spring 1	1	7	*Solea senegalensis*	L	114	21	477	129.7	204.1	122	19.3	0.507	0.335	0.372
Spring 1	1	8	*Solea senegalensis*	M	170	26	104	0.0	3.2	0.8	4.2	0.000	0.005	0.002
Spring 1	1	8	*Solea senegalensis*	L	170	26	39	0.8	31.3	0.5	1.6	0.003	0.051	0.001
Spring 1	1	8	*Solea senegalensis*	G	170	26	12	3.5	14.4	5.2	0.5	0.014	0.024	0.016
Spring 1	1	9	*Solea senegalensis*	L	149	25	200	3.1	40.0	1.4	8.1	0.012	0.066	0.004
Spring 1	1	9	*Solea senegalensis*	G	149	25	18	1.8	20.1	3.1	0.7	0.007	0.033	0.010
Spring 1	1	9	*Solea senegalensis*	M	149	25	179	2.4	2.2	0.3	7.2	0.009	0.004	0.001
Spring 1	1	10	*Solea senegalensis*	M	153	26	126	1.9	2.9	2.7	5.1	0.008	0.005	0.008
Spring 1	1	10	*Solea senegalensis*	G	153	26	15	10.2	18.1	12.5	0.6	0.040	0.030	0.038
Spring 1	1	11	*Solea senegalensis*	M	183	28	123	0.7	3.1	1.7	5.0	0.003	0.005	0.005
Spring 1	1	11	*Solea senegalensis*	L	183	28	148	1.1	33.1	0.9	6.0	0.004	0.054	0.003
Spring 1	1	11	*Solea senegalensis*	G	183	28	19	2.2	20.9	4.9	0.8	0.009	0.034	0.015
Spring 1	1	12	*Solea senegalensis*	G	112	23	10	n.d.	15.6	2.7	0.4	n.d.	0.026	0.008
Spring 1	1	12	*Solea senegalensis*	M	112	23	52	n.d.	0.5	0.3	2.1	n.d.	0.001	0.001
Spring 1	1	13	*Solea senegalensis*	G	130	25	9	8.3	10.9	15.2	0.4	0.032	0.018	0.047
Spring 1	1	13	*Solea senegalensis*	M	130	25	74	0.8	1.8	2.8	3.0	0.003	0.003	0.008
Spring 1	1	13	*Solea senegalensis*	L	130	25	25	1.1	8.6	0.1	1.0	0.004	0.014	0.000
Spring 1	1	14	*Solea senegalensis*	L	134	24	34	0.3	14.1	0.4	1.4	0.001	0.023	0.001
Spring 1	1	14	*Solea senegalensis*	M	134	24	84	n.d.	0.5	0.0	3.4	n.d.	0.001	0.000
Spring 1	1	14	*Solea senegalensis*	G	134	24	14	0.9	17.0	3.3	0.6	0.004	0.028	0.010
Spring 1	1	15	*Solea senegalensis*	M	274	31	153	2.2	2.3	1.2	6.2	0.009	0.004	0.004
Spring 1	1	15	*Solea senegalensis*	G	274	31	19	9.2	34.0	5.0	0.7	0.036	0.056	0.015
Spring 1	1	16	*Solea senegalensis*	G	199	28	8	2.8	9.6	4.7	0.3	0.011	0.016	0.014
Spring 1	1	16	*Solea senegalensis*	L	199	28	35	0.7	16.1	0.6	1.4	0.003	0.026	0.002
Spring 1	1	16	*Solea senegalensis*	M	199	28	65	0.4	1.9	2.7	2.6	0.002	0.003	0.008
Spring 1	1	2	*Diplodus sargus sargus*	M	77	7.5	60	n.d.	0.9	n.d.	2.4	n.d.	0.001	n.d.
Spring 1	1	2	*Diplodus sargus sargus*	L	77	7.5	40	1.7	45.3	0.5	1.6	0.007	0.074	0.001
Spring 1	1	2	*Diplodus sargus sargus*	G	77	7.5	28	2.3	15.3	3.1	1.1	0.009	0.025	0.009
Spring 1	1	17	*Solea senegalensis*	M	223	29	159	1.0	4.6	3.3	6.4	0.004	0.008	0.010
Spring 1	1	17	*Solea senegalensis*	L	223	29	188	0.4	19.8	0.1	7.6	0.002	0.032	0.000
Spring 1	1	17	*Solea senegalensis*	G	223	29	19	8.5	19.3	3.2	0.8	0.033	0.032	0.010
Spring 1	1	18	*Solea senegalensis*	G	201	28	46	3.8	10.8	7.8	1.8	0.015	0.018	0.024
Spring 1	1	18	*Solea senegalensis*	M	201	28	108	0.0	1.3	1.9	4.3	0.000	0.002	0.006
Spring 1	1	18	*Solea senegalensis*	L	201	28	292	3.3	34.7	0.7	11.8	0.013	0.057	0.002
Spring 1	2	19	*Solea senegalensis*	L	242	28	372	1.3	2.3	0.1	10.7	0.005	0.012	0.000
Spring 1	2	19	*Solea senegalensis*	M	242	28	171	n.d.	0.3	0.1	4.9	n.d.	0.002	0.000
Spring 1	2	19	*Solea senegalensis*	G	242	28	25	9.1	10.6	9.3	0.7	0.037	0.056	0.028
Spring 1	2	20	*Solea senegalensis*	M	287	32	102	1.0	1.0	1.4	2.9	0.004	0.005	0.004
Spring 1	2	20	*Solea senegalensis*	G	287	32	23	2.4	2.7	1.3	0.7	0.010	0.014	0.004
Spring 1	2	21	*Solea senegalensis*	M	386	37	366	n.d.	0.4	0.4	10.5	n.d.	0.002	0.001
Spring 1	2	21	*Solea senegalensis*	G	386	37	98	2.4	7.0	1.5	2.8	0.010	0.037	0.004
Spring 1	3	22	*Solea senegalensis*	G	197	28	52	2.3	11.4	6.5	4.4	0.003	0.017	0.004
Spring 1	3	22	*Solea senegalensis*	L	197	28	133	0.3	10.2	0.2	11.3	0.000	0.015	0.000
Spring 1	3	22	*Solea senegalensis*	M	197	28	314	n.d.	0.1	0.1	26.6	n.d.	0.000	0.000
Spring 1	3	3	*Trigloporus lastoviza*	G	339	35	17	1.5	16.5	1.1	1.4	0.002	0.024	0.001
Spring 1	3	3	*Trigloporus lastoviza*	L	339	35	24	0.4	93.4	0.1	2.0	0.001	0.136	0.000
Spring 1	3	3	*Trigloporus lastoviza*	M	339	35	22	1.9	2.2	0.4	1.9	0.002	0.003	0.000
Spring 1	3	4	*Trigloporus lastoviza*	L	400	37	172	0.3	50.9	0.7	14.6	0.000	0.074	0.000
Spring 1	3	4	*Trigloporus lastoviza*	G	400	37	89	1.6	18.4	1.3	7.5	0.002	0.027	0.001
Spring 1	3	4	*Trigloporus lastoviza*	M	400	37	66	n.d.	4.4	0.2	5.6	n.d.	0.006	0.000
Spring 1	3	5	*Trigloporus lastoviza*	G	371	34	91	2.8	28.7	4.9	7.7	0.003	0.042	0.003
Spring 1	3	5	*Trigloporus lastoviza*	M	371	34	99	n.d.	5.7	0.7	8.4	n.d.	0.008	0.000
Spring 1	3	5	*Trigloporus lastoviza*	M	371	38	199	0.4	128.8	0.1	16.9	0.001	0.188	0.000
Spring 1	3	6	*Trigloporus lastoviza*	L	327	32	41	1.1	62.6	0.7	3.5	0.001	0.091	0.000
Spring 1	3	6	*Trigloporus lastoviza*	M	327	32	72	0.1	6.1	0.4	6.1	0.000	0.009	0.000
Spring 1	3	6	*Trigloporus lastoviza*	G	327	32	39	2.6	27.0	2.6	3.3	0.003	0.039	0.002
Spring 1	3	7	*Trigloporus lastoviza*	M	353	34	13	0.1	2.9	0.2	1.1	0.000	0.004	0.000
Spring 1	3	7	*Trigloporus lastoviza*	G	353	34	13	2.8	21.3	4.0	1.1	0.003	0.031	0.003
Spring 1	3	7	*Trigloporus lastoviza*	L	353	32	15	1.9	168.5	0.0	1.3	0.002	0.246	0.000
Spring 1	3	8	*Trigloporus lastoviza*	L	291	31	22	0.6	78.3	0.2	1.8	0.001	0.114	0.000
Spring 1	3	8	*Trigloporus lastoviza*	M	291	31	29	n.d.	4.2	0.3	2.5	n.d.	0.006	0.000
Spring 1	3	8	*Trigloporus lastoviza*	G	291	31	28	4.9	26.0	9.2	2.3	0.006	0.038	0.006
Spring 1	3	9	*Trigloporus lastoviza*	M	319	34	82	0.1	2.1	0.4	6.9	0.000	0.003	0.000
Spring 1	3	9	*Trigloporus lastoviza*	L	319	34	44	0.7	134.5	0.1	3.7	0.001	0.196	0.000
Spring 1	3	9	*Trigloporus lastoviza*	G	319	34	42	2.2	19.6	2.3	3.5	0.003	0.029	0.002
Spring 1	3	10	*Trigloporus lastoviza*	M	348	33	70	n.d.	2.3	0.2	5.9	n.d.	0.003	0.000
Spring 1	3	10	*Trigloporus lastoviza*	L	348	33	51	0.1	42.4	n.d.	4.3	0.000	0.062	n.d.
Spring 1	3	10	*Trigloporus lastoviza*	G	348	33	50	2.7	20.5	2.2	4.3	0.003	0.030	0.002
Spring 1	3	11	*Trigloporus lastoviza*	M	400	39	62	n.d.	4.9	0.6	5.2	n.d.	0.007	0.000
Spring 1	3	11	*Trigloporus lastoviza*	L	400	39	117	0.1	121.8	0.2	9.9	0.000	0.178	0.000
Spring 1	3	11	*Trigloporus lastoviza*	G	400	39	48	3.4	29.9	3.1	4.1	0.004	0.044	0.002
Spring 1	3	12	*Trigloporus lastoviza*	L	306	32	31	0.5	57.9	0.2	2.6	0.001	0.084	0.000
Spring 1	3	12	*Trigloporus lastoviza*	G	306	32	25	1.8	22.9	3.5	2.1	0.002	0.033	0.002
Spring 1	3	12	*Trigloporus lastoviza*	M	306	32	53	n.d.	5.3	0.9	4.5	n.d.	0.008	0.001
Spring 1	3	13	*Trigloporus lastoviza*	G	290	38	34	4.1	35.4	6.6	2.9	0.005	0.052	0.005
Spring 1	3	13	*Trigloporus lastoviza*	L	290	38	26	1.1	91.9	0.2	2.2	0.001	0.134	0.000
Spring 1	3	13	*Trigloporus lastoviza*	M	290	38	34	1.0	4.0	0.2	2.8	0.001	0.006	0.000
Spring 1	3	14	*Trigloporus lastoviza*	M	299	32	50	n.d.	2.9	0.3	4.2	n.d.	0.004	0.000
Spring 1	3	14	*Trigloporus lastoviza*	L	299	32	57	0.7	69.1	0.3	4.8	0.001	0.101	0.000
Spring 1	3	14	*Trigloporus lastoviza*	G	299	32	41	1.5	21.6	2.1	3.4	0.002	0.031	0.001
Spring 1	4	23	*Solea senegalensis*	M	213	28	163	0.3	1.6	1.3	11.0	0.000	0.003	0.002
Spring 1	4	23	*Solea senegalensis*	L	213	28	67	1.0	8.9	0.3	4.5	0.001	0.016	0.000
Spring 1	4	23	*Solea senegalensis*	G	213	28	19	1.6	10.4	0.7	1.3	0.002	0.018	0.001
Spring 1	5	24	*Solea senegalensis*	G	235	29	141	2.0	101.6	5.9	11.4	0.002	0.583	0.002
Spring 1	5	24	*Solea senegalensis*	L	235	29	136	0.3	3.0	2.3	11.0	0.000	0.017	0.001
Spring 1	5	24	*Solea senegalensis*	M	235	29	205	n.d.	0.3	1.8	16.6	n.d.	0.001	0.001
Spring 1	5	25	*Solea senegalensis*	L	172	26	49	n.d.	3.7	0.6	4.0	n.d.	0.021	0.000
Spring 1	5	25	*Solea senegalensis*	G	172	26	15	0.4	2.9	5.1	1.2	0.000	0.017	0.002
Spring 1	5	25	*Solea senegalensis*	M	172	26	199	0.1	0.2	2.1	16.1	0.000	0.001	0.001
Autumn 2	1	26	*Solea senegalensis*	L	174	25	44	1.1	8.1	n.d.	2.7	0.006	0.022	n.d.
Autumn 2	1	26	*Solea senegalensis*	G	174	25	14	0.2	11.5	1.2	0.9	0.001	0.032	0.004
Autumn 2	1	26	*Solea senegalensis*	M	174	25	90	0.2	3.7	0.7	5.6	0.001	0.010	0.002
Autumn 2	1	27	*Solea senegalensis*	L	150	25	55	1.8	21.3	0.6	3.4	0.011	0.059	0.002
Autumn 2	1	27	*Solea senegalensis*	M	150	25	88	n.d.	1.9	1.5	5.5	n.d.	0.005	0.005
Autumn 2	1	28	*Solea senegalensis*	L	256	31	121	2.4	13.7	n.d.	7.6	0.014	0.038	n.d.
Autumn 2	1	28	*Solea senegalensis*	M	256	31	194	0.4	0.9	0.3	12.1	0.002	0.002	0.001
Autumn 2	1	28	*Solea senegalensis*	G	256	31	5	2.1	18.3	9.3	0.3	0.013	0.051	0.030
Autumn 2	1	29	*Solea senegalensis*	M	237	29	97	2.3	3.2	2.4	6.0	0.014	0.009	0.008
Autumn 2	1	29	*Solea senegalensis*	G	237	29	11	4.2	11.0	0.2	0.7	0.025	0.031	0.001
Autumn 2	1	1	*Scorpaena porcus*	L	>440	50	54	1.4	18.4	0.2	3.4	0.008	0.051	0.001
Autumn 2	1	30	*Solea senegalensis*	M	223	29	122	0.1	4.5	1.1	7.6	0.000	0.012	0.003
Autumn 2	1	30	*Solea senegalensis*	G	223	29	12	0.4	15.0	2.6	0.7	0.002	0.042	0.009
Autumn 2	1	2	*Scorpaena porcus*	L	345	26	34	0.7	69.5	n.d.	2.2	0.004	0.193	n.d.
Autumn 2	1	2	*Scorpaena porcus*	G	345	26	7	2.6	19.3	1.5	0.4	0.016	0.054	0.005
Autumn 2	1	2	*Scorpaena porcus*	G	345	26	13	2.7	17.3	3.4	0.8	0.016	0.048	0.011
Autumn 2	1	2	*Scorpaena porcus*	M	345	26	21	n.d.	2.2	2.5	1.3	n.d.	0.006	0.008
Autumn 2	1	15	*Trigloporus lastoviza*	G	261	29	8	0.0	12.2	n.d.	0.5	0.000	0.034	n.d.
Autumn 2	1	15	*Trigloporus lastoviza*	L	261	29	27	1.2	35.8	0.0	1.7	0.007	0.099	0.000
Autumn 2	1	3	*Scorpaena porcus*	L	206	20	11	0.1	12.7	n.d.	0.7	0.000	0.035	n.d.
Autumn 2	1	3	*Scorpaena porcus*	G	206	20	7	1.7	15.0	0.5	0.4	0.010	0.042	0.002
Autumn 2	1	3	*Scorpaena porcus*	M	206	20	43	n.d.	2.5	0.1	2.7	n.d.	0.007	0.000
Autumn 2	1	31	*Solea senegalensis*	L	348	35	67	2.5	11.2	0.0	4.2	0.015	0.031	0.000
Autumn 2	1	31	*Solea senegalensis*	G	348	35	5	0.3	9.5	0.9	0.3	0.002	0.026	0.003
Autumn 2	1	31	*Solea senegalensis*	M	348	35	118	n.d.	0.3	0.5	7.4	n.d.	0.001	0.001
Autumn 2	1	4	*Scorpaena porcus*	L	324	25	14	0.1	16.4	n.d.	0.9	0.001	0.045	n.d.
Autumn 2	1	4	*Scorpaena porcus*	G	324	25	9	1.0	15.5	0.2	0.6	0.006	0.043	0.001
Autumn 2	1	4	*Scorpaena porcus*	M	324	25	19	0.2	5.0	3.3	1.2	0.001	0.014	0.011
Autumn 2	1	4	*Scorpaena porcus*	M	324	25	19	1.0	2.5	1.6	1.2	0.006	0.007	0.005
Autumn 2	1	5	*Scorpaena porcus*	M	>440	34	10	0.4	6.2	1.1	0.6	0.002	0.017	0.004
Autumn 2	1	5	*Scorpaena porcus*	M	>440	34	17	0.1	2.9	0.5	1.1	0.001	0.008	0.002
Autumn 2	1	5	*Scorpaena porcus*	G	>440	34	7	1.1	10.2	0.4	0.5	0.006	0.028	0.001
Autumn 2	2	6	*Scorpaena porcus*	L	>440	31	44	0.2	21.2	n.d.	0.7	0.001	0.066	n.d.
Autumn 2	2	6	*Scorpaena porcus*	M	>440	31	42	n.d.	2.0	1.6	0.7	n.d.	0.006	0.003
Autumn 2	2	6	*Scorpaena porcus*	G	>440	31	35	0.7	9.2	1.4	0.6	0.001	0.028	0.003
Autumn 2	2	7	*Scorpaena porcus*	L	284	28	32	2.1	33.5	n.d.	0.5	0.004	0.104	n.d.
Autumn 2	2	7	*Scorpaena porcus*	M	234	28	94	1.1	7.6	1.0	1.6	0.002	0.024	0.002
Autumn 2	2	7	*Scorpaena porcus*	G	234	28	50	4.4	21.1	0.9	0.8	0.009	0.065	0.002
Autumn 2	2	8	*Scorpaena porcus*	M	341	26	69	n.d.	3.0	0.6	1.2	n.d.	0.009	0.001
Autumn 2	2	8	*Scorpaena porcus*	L	293	24	19	0.5	20.1	n.d.	0.3	0.001	0.062	n.d.
Autumn 2	2	8	*Scorpaena porcus*	G	293	24	19	3.8	12.8	0.7	0.3	0.008	0.039	0.001
Autumn 2	2	9	*Scorpaena porcus*	L	341	28	61	1.3	118.8	0.2	1.0	0.003	0.367	0.000
Autumn 2	2	9	*Scorpaena porcus*	G	341	26	49	1.5	3.4	0.0	0.8	0.003	0.011	0.000
Autumn 2	3	16	*Trigloporus lastoviza*	M	175	26	39	0.1	1.7	0.6	2.3	0.000	0.007	0.000
Autumn 2	3	16	*Trigloporus lastoviza*	G	175	26	24	2.4	5.4	1.4	1.4	0.005	0.023	0.001
Autumn 2	3	32	*Solea senegalensis*	L	196	26	58	0.6	2.4	0.1	3.4	0.001	0.010	0.000
Autumn 2	3	32	*Solea senegalensis*	M	196	26	143	0.1	1.0	0.4	8.5	0.000	0.004	0.000
Autumn 2	3	33	*Solea senegalensis*	L	745	20	36	1.9	3.1	1.0	2.2	0.004	0.013	0.001
Autumn 2	3	33	*Solea senegalensis*	G	75	20	8	2.0	4.6	9.1	0.5	0.004	0.019	0.008
Autumn 2	3	33	*Solea senegalensis*	M	75	20	37	0.0	0.3	0.9	2.2	0.000	0.001	0.001
Autumn 2	3	17	*Trigloporus lastoviza*	L	177	27	45	1.4	49.9	n.d.	2.7	0.003	0.209	n.d.
Autumn 2	3	17	*Trigloporus lastoviza*	G	177	27	n.d.	n.d.	n.d.	n.d.	n.d.	n.d.	n.d.	n.d.
Autumn 2	3	17	*Trigloporus lastoviza*	M	177	27	63	n.d.	0.8	0.6	3.8	n.d.	0.003	0.001
Autumn 2	3	34	*Solea senegalensis*	L	148	26	112	2.7	8.8	2.8	6.6	0.006	0.037	0.002
Autumn 2	3	34	*Solea senegalensis*	G	148	26	20	3.6	3.5	0.7	1.2	0.008	0.015	0.001
Autumn 2	3	34	*Solea senegalensis*	M	148	26	88	0.1	0.6	1.5	5.2	0.000	0.003	0.001
Autumn 2	3	34	*Solea senegalensis*	M	217	30	n.d.	n.d.	n.d.	n.d.	n.d.	n.d.	n.d.	n.d.
Autumn 2	3	35	*Solea senegalensis*	G	217	30	19	0.7	4.0	2.5	1.1	0.002	0.017	0.002
Autumn 2	3	35	*Solea senegalensis*	L	217	30	65	2.7	23.7	n.d.	3.9	0.006	0.099	n.d.
Autumn 2	4	18	*Trigloporus lastoviza*	M	175	26	107	n.d.	0.4	8.3	2.9	n.d.	0.004	0.009
Autumn 2	4	18	*Trigloporus lastoviza*	L	175	26	181	6.0	40.7	0.5	4.8	0.015	0.413	0.001
Autumn 2	4	18	*Trigloporus lastoviza*	G	175	26	82	1.0	3.3	0.7	2.2	0.002	0.034	0.001
Autumn 2	4	10	*Scorpaena porcus*	L	108	18	23	0.6	1.5	0.1	0.6	0.002	0.016	0.000
Autumn 2	4	10	*Scorpaena porcus*	G	133	19	22	2.0	2.3	0.8	0.6	0.005	0.024	0.001
Autumn 2	4	10	*Scorpaena porcus*	M	133	19	263	n.d.	0.7	0.6	7.0	n.d.	0.007	0.001
Autumn 2	4	11	*Scorpaena porcus*	M	108	18	228	n.d.	0.6	1.1	6.1	n.d.	0.006	0.001
Autumn 2	4	11	*Scorpaena porcus*	G	108	18	22	0.8	2.3	1.3	0.6	0.002	0.024	0.001
Autumn 2	4	19	*Trigloporus lastoviza*	M	135	23	83	n.d.	0.5	1.8	2.2	n.d.	0.005	0.002
Autumn 2	4	19	*Trigloporus lastoviza*	L	135	23	102	1.0	17.1	n.d.	2.7	0.002	0.174	n.d.
Autumn 2	4	19	*Trigloporus lastoviza*	G	135	23	77	1.5	4.3	2.1	2.1	0.004	0.044	0.002
Autumn 2	4	20	*Trigloporus lastoviza*	M	135	24	144	n.d.	0.8	0.4	3.8	n.d.	0.009	0.000
Autumn 2	4	20	*Trigloporus lastoviza*	G	135	24	62	0.9	6.6	0.0	1.7	0.002	0.067	0.000
Autumn 2	4	20	*Trigloporus lastoviza*	L	135	23	88	2.2	0.5	n.d.	2.3	0.005	0.005	n.d.
Autumn 2	5	21	*Trigloporus lastoviza*	L	124	23	257	6.6	1003.3	0.5	7.2	0.007	0.943	0.001
Autumn 2	5	21	*Trigloporus lastoviza*	G	124	23	67	3.9	124.1	1.0	1.9	0.004	0.117	0.002
Autumn 2	5	37	*Solea senegalensis*	G	>440	36	9	2.7	25.7	0.4	0.2	0.003	0.024	0.001
Autumn 2	5	36	*Solea senegalensis*	M	>440	36	97	1.0	2.1	0.8	2.7	0.001	0.002	0.001
Autumn 2	5	36	*Solea senegalensis*	L	>440	36	90	4.2	11.2	0.2	2.5	0.004	0.011	0.000
Autumn 2	5	37	*Solea senegalensis*	M	43	35	392	n.d.	2.4	3.0	11.0	n.d.	0.002	0.005
Autumn 2	5	37	*Solea senegalensis*	G	43	35	40	9.1	39.3	5.2	1.1	0.010	0.037	0.008
Autumn 2	5	37	*Solea senegalensis*	L	43	35	25	1.0	71.2	0.2	0.7	0.001	0.067	0.000
Autumn 2	5	38	*Solea senegalensis*	M	137	28	148	7.8	2.5	0.5	4.2	0.008	0.002	0.001
Autumn 2	5	38	*Solea senegalensis*	G	137	28	30	6.6	22.4	2.7	0.8	0.007	0.021	0.004
Spring 2	1	39	*Solea senegalensis*	G	193	26	7	3.9	2.4	1.1	0.3	0.018	0.009	0.003
Spring 2	1	39	*Solea senegalensis*	M	193	26	64	1.4	0.6	2.5	2.7	0.007	0.002	0.007
Spring 2	1	39	*Solea senegalensis*	L	193	26	15	0.3	2.8	0.4	0.6	0.001	0.010	0.001
Spring 2	1	40	*Solea senegalensis*	M	193	26	115	2.4	1.2	1.4	4.9	0.011	0.004	0.004
Spring 2	1	40	*Solea senegalensis*	G	193	26	10	4.9	5.4	1.9	0.4	0.023	0.019	0.006
Spring 2	1	40	*Solea senegalensis*	L	193	26	42	0.8	5.8	0.2	1.8	0.004	0.021	0.001
Spring 2	1	41	*Solea senegalensis*	G	130	24	9	5.1	2.5	2.2	0.4	0.024	0.009	0.006
Spring 2	1	41	*Solea senegalensis*	L	130	24	27	2.6	5.0	2.1	1.1	0.012	0.018	0.006
Spring 2	1	41	*Solea senegalensis*	M	130	24	91	0.7	0.3	1.7	3.9	0.003	0.001	0.005
Spring 2	1	42	*Solea senegalensis*	M	122	25	111	0.8	0.5	0.4	4.7	0.004	0.002	0.001
Spring 2	1	42	*Solea senegalensis*	L	122	25	19	1.9	2.8	0.7	0.8	0.009	0.010	0.002
Spring 2	1	42	*Solea senegalensis*	G	122	25	8	3.1	1.6	10.6	0.3	0.015	0.005	0.031
Spring 2	1	43	*Solea senegalensis*	G	166	26	4	11.0	4.3	4.8	0.2	0.052	0.015	0.014
Spring 2	1	43	*Solea senegalensis*	M	166	26	68	1.7	0.7	2.5	2.9	0.008	0.003	0.007
Spring 2	1	43	*Solea senegalensis*	L	166	26	26	0.7	5.5	n.d.	1.1	0.003	0.019	n.d.
Spring 2	1	44	*Solea senegalensis*	G	117	23	1	0.8	0.6	0.8	0.0	0.004	0.002	0.002
Spring 2	1	44	*Solea senegalensis*	M	117	23	87	1.1	0.4	0.7	3.7	0.005	0.002	0.002
Spring 2	1	44	*Solea senegalensis*	L	117	23	46	1.5	55.8	2.4	1.7	0.007	0.194	0.005
Spring 2	1	45	*Solea senegalensis*	M	82	20	62	1.9	1.0	1.6	2.6	0.009	0.004	0.005
Spring 2	1	45	*Solea senegalensis*	G	82	20	6	9.9	5.9	11.1	0.3	0.046	0.021	0.033
Spring 2	1	45	*Solea senegalensis*	L	82	20	2	0.3	1.6	0.0	0.1	0.000	0.003	0.000
Spring 2	1	12	*Scorpaena porcus*	M	149	20	21	1.6	1.0	1.8	0.9	0.007	0.004	0.005
Spring 2	1	12	*Scorpaena porcus*	G	149	20	7	2.9	5.4	1.5	0.3	0.014	0.019	0.004
Spring 2	1	12	*Scorpaena porcus*	L	149	20	22	0.3	23.0	0.3	0.9	0.001	0.081	0.001
Spring 2	1	3	*Diplodus sargus sargus*	G	149	20	11	1.5	4.7	1.8	0.4	0.007	0.017	0.005
Spring 2	1	3	*Diplodus sargus sargus*	M	149	20	10	2.0	0.4	1.2	0.4	0.010	0.001	0.003
Spring 2	1	3	*Diplodus sargus sargus*	L	149	20	20	2.1	28.1	n.d.	0.9	0.010	0.099	n.d.
Spring 2	2	4	*Diplodus sargus sargus*	L	60	36	50	3.4	115.9	0.3	1.8	0.020	0.278	0.001
Spring 2	2	4	*Diplodus sargus sargus*	M	60	36	64	1.5	4.1	0.7	2.0	0.009	0.010	0.002
Spring 2	2	4	*Diplodus sargus sargus*	G	60	36	23	3.1	25.7	1.2	0.7	0.018	0.061	0.004
Spring 2	2	5	*Diplodus sargus sargus*	M	153	21	54	1.1	3.4	0.7	1.7	0.006	0.008	0.002
Spring 2	2	5	*Diplodus sargus sargus*	G	153	21	23	2.5	17.4	1.8	0.7	0.014	0.041	0.006
Spring 2	2	5	*Diplodus sargus sargus*	L	153	21	18	1.4	43.2	0.1	0.6	0.008	0.102	0.000
Spring 2	4	46	*Solea senegalensis*	G	202	27	9	3.3	3.6	5.0	0.3	0.009	0.009	0.005
Spring 2	4	46	*Solea senegalensis*	M	202	27	112	1.2	0.5	1.4	4.2	0.003	0.001	0.001
Spring 2	4	46	*Solea senegalensis*	L	202	27	38	0.1	5.7	0.1	1.4	0.000	0.015	0.000
Spring 2	4	47	*Solea senegalensis*	G	79	20	47	7.8	3.5	2.6	1.7	0.021	0.009	0.003
Spring 2	4	47	*Solea senegalensis*	L	79	20	22	0.1	1.1	n.d.	0.8	0.000	0.003	n.d.
Spring 2	4	22	*Trigloporus lastoviza*	L	113	22	21	1.4	171.5	0.1	0.7	0.014	0.894	0.000
Spring 2	4	22	*Trigloporus lastoviza*	G	113	22	15	3.0	22.4	4.5	0.6	0.008	0.059	0.004
Spring 2	4	22	*Trigloporus lastoviza*	M	113	22	20	0.7	2.3	2.0	0.7	0.002	0.006	0.002
Spring 2	4	13	*Scorpaena porcus*	G	118	17	6	3.6	8.1	2.3	0.2	0.010	0.021	0.002
Spring 2	4	13	*Scorpaena porcus*	M	118	17	48	1.3	5.0	0.7	1.8	0.003	0.013	0.001
Spring 2	4	13	*Scorpaena porcus*	L	118	17	9	1.2	11.6	0.2	0.3	0.003	0.030	0.000
Spring 2	4	14	*Scorpaena porcus*	G	88	16	6	1.7	11.1	2.3	0.2	0.005	0.029	0.002
Spring 2	4	14	*Scorpaena porcus*	M	88	16	38	1.0	2.6	0.8	1.4	0.003	0.007	0.001
Spring 2	4	15	*Scorpaena porcus*	M	101	17	39	0.3	4.8	0.8	1.4	0.001	0.013	0.001
Spring 2	4	15	*Scorpaena porcus*	G	101	17	7	1.8	13.5	1.5	0.3	0.005	0.035	0.001
Spring 2	4	15	*Scorpanea porcus*	L	101	17	17	2.3	31.6	0.2	0.6	0.006	0.083	0.000

**Table A2:** Limits of detection (μg/L) for As, Ni, Co and Cr in water, sediment and fish samples (n = 10).

Samples	Metal			
	As	Ni	Co	Cr
Water	0.098	0.007	0.004	0.007
Total metal in sediments	0.444	0.379	0.101	0.712
Acid-Extractable fraction in sediments	0.269	0.454	0.068	0.727
Reducible fraction in sediments	0.002	1.281	0.001	1.345
Oxidisable fraction in sediments	0.618	1.242	0.118	0.297
Residual fraction in sediments	2.284	0.286	0.105	0.829
Liver	0.505	0.243	0.025	0.139
Gills and Muscle	0.472	0.552	0.084	0.285

**Table A3:** Concentrations of As (mg/kg) and Ni (mg/kg) in the different fractions of sediments from Algeciras Bay.

Samplings	Sites	As (mg/kg)				Ni (mg/kg)			
		Acid Extractable Fraction	Reducible Fraction	Oxidisable Fraction	Residual Fraction	Acid Extractable Fraction	Reducible Fraction	Oxidisable Fraction	Residual Fraction
autumn 1 (1st)	1	0.233	1.045	0.925	12.870	0.709	0.303	1.458	36.872
	2	0.488	0.764	1.583	35.780	0.704	0.730	2.890	91.774
	3	0.368	0.904	0.400	4.422	10.406	14.863	10.354	72.646
	4	0.317	1.941	0.698	1.490	4.044	11.241	10.710	77.995
	5	0.431	1.229	0.953	7.724	0.961	0.699	4.047	88.090
spring 1 (2nd)	1	0.317	0.676	0.899	7.103	0.438	0.000	5.217	25.615
	2	0.497	1.862	1.024	21.063	0.574	0.034	1.612	65.617
	3	0.140	0.805	0.200	1.216	4.856	9.990	8.361	134.311
	4	0.307	0.591	0.304	0.146	2.902	10.474	9.222	49.966
	5	0.208	1.305	0.356	1.488	1.770	18.511	11.230	65.681
autumn 2 (3rd)	1	0.313	0.624	0.719	16.836	1.124	0.722	2.221	55.434
	2	0.448	0.647	0.942	27.740	1.266	0.161	2.770	98.673
	3	0.248	1.242	0.227	4.295	3.486	10.082	8.404	79.653
	4	0.175	1.856	0.465	7.452	2.524	10.271	10.030	75.665
	5	0.446	1.631	0.674	10.575	2.427	10.151	7.547	69.671
spring 2 (4th)	1	0.325	0.766	0.594	10.924	1.155	1.397	2.055	44.634
	2	0.467	0.766	0.852	22.696	1.435	0.145	1.477	66.280
	3	0.228	0.920	0.144	5.994	3.341	5.291	6.261	51.933
	4	0.266	0.911	0.175	2.398	1.473	11.889	7.522	45.298
	5	0.292	1.333	0.492	7.652	1.650	9.085	7.952	56.753

**Table A4:** Concentrations of Co (mg/kg) and Cr (mg/kg) in the different fractions of sediments from Algeciras Bay.

Samplings	Sites	Co (mg/kg)				Cr (mg/kg)			
		Acid Extractable Fraction	Reducible Fraction	Oxidisable Fraction	Residual Fraction	Acid Extractable Fraction	Reducible Fraction	Oxidisable Fraction	Residual Fraction
autumn 1 (1st)	1	0.177	0.243	1.392	12.870	0.000	0.412	7.190	63.738
	2	0.912	0.415	3.394	35.780	0.000	0.000	7.702	227.420
	3	4.098	2.881	1.609	4.422	1.355	19.100	24.379	220.103
	4	12.936	6.796	3.800	1.490	0.529	8.054	16.448	206.190
	5	0.835	0.252	1.701	7.724	0.138	0.000	5.604	169.472
spring 1 (2nd)	1	0.469	0.670	2.094	5.934	1.815	0.000	3.392	44.736
	2	0.615	0.184	1.418	11.736	0.000	0.000	7.252	164.573
	3	2.810	2.164	1.273	12.884	0.476	8.145	10.867	344.192
	4	3.931	2.783	1.429	5.133	0.919	7.735	11.117	127.999
	5	1.603	2.627	1.001	6.066	0.733	9.770	8.009	228.244
autumn 2 (3rd)	1	0.271	0.062	1.305	9.776	0.169	0.000	5.332	111.438
	2	0.587	0.106	1.912	13.847	0.122	0.000	8.700	256.171
	3	1.666	2.002	1.282	8.481	0.783	10.256	13.315	206.086
	4	5.432	5.332	3.464	7.385	0.420	10.002	13.554	203.756
	5	0.808	2.036	1.177	8.151	0.265	6.295	8.320	153.956
spring 2 (4th)	1	0.254	0.047	0.847	7.100	0.235	0.000	3.169	89.870
	2	0.413	0.078	1.167	9.986	0.389	0.000	6.185	167.525
	3	1.936	1.861	1.114	8.377	0.712	5.128	9.950	153.534
	4	1.564	1.805	0.746	3.629	0.791	9.137	6.836	148.054
	5	0.469	2.350	0.843	6.224	0.168	7.916	7.848	128.534
